# Analysis of histone post translational modifications in primary monocyte derived macrophages using reverse phase × reverse phase chromatography in conjunction with porous graphitic carbon stationary phase

**DOI:** 10.1016/j.chroma.2016.05.025

**Published:** 2016-07-01

**Authors:** Thomas C. Minshull, Joby Cole, David H. Dockrell, Robert C. Read, Mark J. Dickman

**Affiliations:** a Department of Chemical and Biological Engineering, ChELSI Institute, University of Sheffield, Mappin Street, Sheffield S1 3JD, United Kingdom; b Department of Infection, Immunity & Cardiovascular Disease, University of Sheffield Medical School, United Kingdom; c Sheffield Teaching Hospitals, United Kingdom; d Academic Unit of Clinical and Experimental Sciences, Faculty of Medicine and Institute for Life Sciences, University of Southampton, NIHR Respiratory Biomedical Research Unit, University Hospital Southampton, Southampton SO166YD, United Kingdom

**Keywords:** Histone post translational modifications, Mass spectrometry, Two dimensional chromatography, Porous graphitic stationary phase

## Abstract

A two dimensional-liquid chromatography (2D-LC) based approach was developed for the identification and quantification of histone post translational modifications in conjunction with mass spectrometry analysis. Using a bottom-up strategy, offline 2D-LC was developed using reverse phase chromatography. A porous graphitic carbon stationary phase in the first dimension and a C_18_ stationary phase in the second dimension interfaced with mass spectrometry was used to analyse global levels of histone post translational modifications in human primary monocyte-derived macrophages. The results demonstrated that 84 different histone peptide proteoforms, with modifications at 18 different sites including combinatorial marks were identified, representing an increase in the identification of histone peptides by 65% and 51% compared to two different 1D-LC approaches on the same mass spectrometer.

The use of the porous graphitic stationary phase in the first dimension resulted in efficient separation of histone peptides across the gradient, with good resolution and is orthogonal to the online C_18_ reverse phase chromatography. Overall, more histone peptides were identified using the 2D-LC approach compared to conventional 1D-LC approaches. In addition, a bioinformatic pipeline was developed in-house to enable the high throughput efficient and accurate quantification of fractionated histone peptides. The automation of a section of the downstream analysis pipeline increased the throughput of the 2D-LC–MS/MS approach for the quantification of histone post translational modifications.

## Highlights

• Developed 2D-LC–MS method for the identification and quantification of histone PTMs.
• A porous graphitic carbon stationary phase in the first dimension and a C_18_ stationary phase column in the second dimension interfaced with MS.
• Analysed global levels of histone PTMs in human primary monocyte-derived macrophages.
• 51 different histone peptide proteoforms, including combinatorial marks were identified on histone H3.
• 65% increase in peptides were identified and quantified compared to traditional 1D-LC analysis on the same MS instrument.

## 1 Introduction

Histone post translational modifications (PTMs) play key roles in regulating eukaryotic gene expression. In eukaryotes, DNA exists in a complex with the core histone octamer (a heteromeric tetramer consisting of H3 and H4 proteins and two H2A/H2B dimers) called the nucleosome [1]. Along with H1 and regions of linker DNA this forms chromatin. The N-terminal tail of histones H3 and H4 are modified with a wide range of chemical modifications including acetylation, methylation, ubiquitination, crotonylation and phosphorylation. The “histone code” describes the complex combinatorial PTM patterns that exist on histone tails and less commonly on their globular domain [2]. Histone PTMs are laid down in a dynamic fashion and enzymatic activities exist that deposit and remove particular modifications. Histone N-terminal tails are key targets for PTMs since they protrude from the nucleosome and can make contact with adjacent nucleosomes, thus providing a mechanism for regulating regional protein-DNA and protein–protein interactions. In addition, the PTM of histones provide binding sites for a number of effector molecules that can establish and orchestrate downstream events [3], [4], [5]. Therefore, these histone marks not only dictate chromatin structure but they also control access to the underlying DNA and hence are involved in all DNA based processes including gene expression [3].

Mass spectrometry (MS) has emerged as a powerful method to identify and characterise histone PTMs. MS allows the unbiased identification and quantification of multiple global histone PTMs including combinations in a single experiment [6]. Thus MS is one of the most powerful tools for probing the histone code and the discovery of new histone PTMs. The bottom up approach is the most widely used because of its superior sensitivity, reproducibility and LC separation of isobaric peptides [7]. The enzyme of choice for bottom up analysis is trypsin, due to its low cost, high specificity and efficiency. However, with histone tails having a large number of lysine and arginine residues, use of trypsin generates small hydrophilic peptides that are poorly retained on reverse phase columns. Therefore, a chemical derivatisation step, most commonly by propionic anhydride, is employed. Originally developed by Garcia et al., this technique is now widely employed and results in peptides of suitable length, with greater hydrophobicity, leading to better retention and separation of isobaric peptides on reverse phase columns [8]. Propionylation also reduces the number of isobaric peptides, simplifying the downstream data analysis. However, propionylation is a naturally occurring modification and hence cannot be studied using this technique [9]. Another issue which affects the reliability of both absolute and relative quantification is the impact that modifications have on the ionisation efficiency of peptides [10]. Recently, this issue has been addressed by generating correction factors by normalising the detection efficiencies of 93 synthetic histone peptides [11].

Offline separation of histone proteins is commonly utilised as a way of further simplifying the complex mixture of peptides introduced to the mass spectrometer. This involves the separation of the histone proteins H3, H4 and variants such as H3.1, H3.2 and H3.3. Several methods have been employed including SDS PAGE, TAU-PAGE [12], RP [13], [14] and HILIC [15]. Multidimensional liquid phase chromatographic peptide separations, termed shotgun analyses, have been widely used in proteomics [16], [17], [18], [19], [20], [21], [22], [23]. This technique, although it loses some histone variant information showed comparable identification of histone PTMs and variants [24]. A widely used chromatographic approach involves the orthogonal combination of cation exchange and reverse phase chromatography to separate peptides. Both offline [19], [20] and online workflows [22], [23] have been developed using this methodology. Offline and online workflows have distinct advantages and disadvantages associated with each method. A number of alternative strategies have been developed for shotgun proteome analysis. Two dimensional peptide separations have successfully been developed utilising reverse phase or ion-pair reverse phase chromatography with a different pH in each dimension [25], [26], [27]. Hydrophilic interaction chromatography (HILIC) and zwitterionic HILIC have also been utilised as first dimensions coupled with RP LC–MS/MS analysis [25]. pI based peptide separations using in-solution isoelectric focusing (IEF) of peptides was developed for peptide fractionation and proteome analysis in conjunction with RP LC–MS/MS analysis [26]. In addition, mixed mode (reverse phase-anion exchange chromatography) in conjunction with reverse phase liquid chromatography mass spectrometry analysis has been developed [28].

The application of multidimensional LC–MS/MS has been used in conjunction with bottom up analysis for studying histone PTMs. The separation of the post tryptic and propionylated histone peptides prior to LC–MS/MS using an in-solution isoelectric focusing fractionation in conjunction with traditional approaches outlined above, enabled the identification of 130 unique histone PTM sites, 67 of which were novel [29]. SCX chromatography has also been used as the first dimension in conjunction with 2D LC–MS analysis of histone PTMs [30]. However, there are a number of caveats associated with SCX in the first dimension including the requirement for desalting prior to LC–MS analysis [27]. More recently, the use of porous graphitic carbon (PGC) as a stationary phase in conjunction with reverse-phase chromatography has been used as a first dimension separation prior to LC–MS/MS in proteomics [31]. Furthermore, it has been shown to retain small hydrophobic peptides longer than conventional RP HPLC resulting in increased identifications of these peptides [32]. Therefore, the use of a PGC column in the first dimension was assessed in terms of orthogonality and fractionation efficiency for the separation of histone peptides. The use of this 2D-LC workflow was compared to alternative 1D-LC methods with no prior fractionation, for the identification and quantification of global levels of histone post translational modifications in human primary monocyte derived macrophages (MDMs). Human MDMs are easily obtained and maintained in pure culture over 14 days and are an excellent model of tissue macrophages; cells with a central involvement in innate immune defence and induction of inflammation.

## 2 Materials and methods

### 2.1 Chemicals

Acetonitrile (LC MS grade), trifluoroacetic acid (TFA), formic acid (FA) and water (HPLC grade) were obtained from Fisher Scientific, Loughborough, UK. RPMI 1640 media, l-glutamine and heat inactivated calf serum (HI FCS) were obtained from Lonza. All other chemicals including, trypsin (proteomics grade) and propionic anhydride were purchased from Sigma, Gillingham, UK.

### 2.2 Cell culture

Human monocyte-derived macrophages (MDMs) were isolated from peripheral blood mononuclear cells (PBMCs) from heparinised blood (1 IU of heparin/ml of blood) of healthy donors, with ethical approval from the South Sheffield Research Ethics Committee (07/Q2305/7). The blood was diluted in a 1:1 ratio with sterile phosphate buffered saline (PBS), carefully layered onto lymphocyte separation buffer (1 part separation buffer: 2 parts blood) (Lonza, Basel, Switzerland) and separated by centrifugation (400*g*, 35 min, 20 °C). A proportion of plasma was saved for use in media later, and PBMCs were transferred to a separate tube. Cells were then pelleted (600 rcf, 15 min, 4 °C) and washed in ice cold PBS twice (600 rcf, 5 min, 4 °C). Cells were then placed in RPMI 1640 media (Lonza, Basel, Switzerland) with 20% plasma and 2 mM l-glutamine (Lonza, Basel, Switzerland), and counted using an improved NeuBauer chamber. Isolated PBMCs were seeded in a 6 well plate at a concentration of 2 × 10^6 cells/well in a total of 3 ml of media. Cells were then incubated for 24 h at 37 °C 5% CO_2_ at which point the cells were washed to remove non adherent cells with RPMI 1640 with 10% HI FCS media. Media was replenished every 3–4 days until day 14 when monocytes had differentiated into monocytes derived macrophages (MDMs). Each well was then washed with PBS to remove any dead cells or contaminating lymphocytes and pelleted at 600 rcf, 7 min, 4 °C. Supernatant was then removed and cells flash frozen in liquid nitrogen and stored at −80 °C.

### 2.3 Acid extraction

Acid extraction of histones was performed on frozen pelleted human monocyte derived macrophages cells essentially as previously described [12]. Following extraction, purity and concentration were determined using SDS-PAGE and Bradford assay respectively.

### 2.4 Propionylation and in-solution digestion

Extracted histone samples were propionylated and digested with trypsin as previously described [8]. Briefly, 10 μl of 100 mM ammonium bicarbonate (ABC) and 5 μl of ammonium hydroxide were added to 10 μg of extracted histone proteins. 10 μl of freshly prepared propionyl reagent (3:1 propionic anhydride: acetonitrile) was then added and adjusted to pH 8 if necessary. Samples were incubated at 51 °C for 20 min and vacuum centrifuged to dryness. Samples were re-suspended in 5 μl of 100 mM ABC and 3 μl of ammonium hydroxide and the procedure was repeated. 50 μl of 60 ng/μl of trypsin in 100 mM ABC was added and left to incubate overnight. The reaction was then quenched by the addition of glacial acetic acid. The samples were dried and the propionylation reaction was carried out two more times to propionylated the newly generated N-terminal peptides.

The propionylated, digested histones extracts were resuspended in 0.1% TFA and directly analysed using LC MS (1D-LC–MS), desalted using HyperSep hypercarb tips (1D-LC MS offline clean-up) or fractionated by HPLC using a PGC stationary phase prior to LC MS analysis (2D-LC–MS).

### 2.5 PGC fractionation

HPLC analysis was performed on a RSLC U3000HPLC (Thermo Fisher Scientific, Hemel Hempstead, UK) using a HyperCarb Column (50 × 2.1 mm, I.D. 5 μm particle size) (Thermo Fisher Scientific, Hemel Hempstead, UK). HPLC Buffers: Buffer A: 3% ACN 0.1% TFA, Buffer B: 97% ACN 0.1% TFA. A linear gradient 3–70% buffer B over 25 min was used at a flow rate of 0.2 ml min^−1^ at 30 °C. Chromatograms were recorded at 214 nm 1 min fractions were collected in a 384 lo-bind deepwell plate, starting at 8 min. Fractions were concentrated to dryness using a vacuum concentrator.

### 2.6 LC ESI MS

Samples were re-suspended in 10 μl of 0.1% TFA and a 5 μl aliquot was injected onto an Acclaim C_18_ PepMap trapping column (300 μm I.D. ×5 mm) (Thermo Fisher Scientific, Hemel Hempstead, UK). An 8 min wash step at 30 μl min^−1^ using 0.1% TFA was used for online desalting or a 1 min loading/wash step for offline desalted samples. Peptide separations were performed using Buffer A: 0.1% formic acid (FA) 3% ACN. Buffer B: 0.1% FA 97% ACN. Linear gradient 3–25% B over 28 min, 25–50% B over 10 min and 50–90 over 0.9 min. Linear gradient 3–25% buffer B over 42 min, 25–50% buffer B over 10 min and 50–90 over 5 min using a 150 mm × 75 μm I.D. PepMap C_18_ column (Thermo Fisher Scientific, Hemel Hempstead, UK) online with a maXis mass spectrometer (Bruker Daltonics, Bremen, Germany) in conjunction with CaptiveSpray ion source (Bruker Daltonics, Bremen, Germany). CaptiveSpray capillary 1400 V, dry gas 3.0 l/min, dry heater 150 °C. MS and MS/MS scans (*m*/*z* 100–2000) were acquired in positive ion mode, no active exclusion, peptide charge state selected +2 + 3 + 4, collision energy 10.0 eV, collision cell RF 1200, ion cooler pre storage pulse 5.0 μs, summation factors for MS/MS 1.6, 0.3 s, isolation widths 3–6 *m*/*z*, resolution MS and MS/MS 40,000 (*m*/*z* 1222). Lock mass calibration was performed using HP 1221.990364.

### 2.7 Data analysis

Data was internally calibrated using the lock mass and .mgf files were generated using Data Analysis 4.1 software with a signal to noise threshold of 100 and an absolute intensity threshold of 5000 and a maximum compound number of 50,000. Data was searched using MASCOT v2.5.1 using Swiss-Prot database under the taxonomy *Homo sapiens* which also included common contaminants and a database using only histone protein sequences and common contaminants. Fixed modifications were set as propionyl (N-term and K) Variable modifications were set as acetyl (K), methylpropionyl, dimethyl, and trimethyl (K) phosphoryl (ST). The databases were searched using the following parameters (analysis peptide tolerance = ±0.015 Da, MS/MS tolerance = ±0.1 Da and peptide charge 1+, 2+, 3+ and 4+) using single modifications and combination of modifications. Results were filtered using a fixed FDR <1% in mascot prior to manual verification. For searches using acetyl (K) alone or in combination typical FDR were set >1% to include the H4 4-17 peptide due to high score spectra that matched with a decoy peptide KAGGKGLGKGGKGR. All MS/MS spectra from modified peptides were manually inspected for accurate mass, correct fragment assignment and retention time profiles. All MS/MS spectra for modified sites/peptides identified in this study are shown in Supplementary information. The relative abundance of histone peptides was determined by the integration of smoothed (gauss algorithm, 1 cycle) extracted ion chromatograms (EIC). Quantification was performed using the in house developed Histomatic VBscript which interacted with Data Analysis to generate a specified list of extracted ion chromatograms. Briefly, Extracted ion chromatograms for each of the manually verified peptides were generated using a tolerance of 0.025 Da. The peaks corresponding to the peptides were smoothed using a Gaussian algorithm integrated to determine the area under the curve (AUC). The use of smoothing does not affect the accuracy of quantification as shown in supplementary Fig. 1. Correction factor values were then applied to each peptide to take into account differences in ionisation efficiency [11]. This value was then used to determine relative abundances for each peptide, by dividing the corrected AUC of one proteoform of a peptide by the sum total of the corrected AUC of all forms of that peptide.

## 3 Results & discussion

**Fig. 1:**
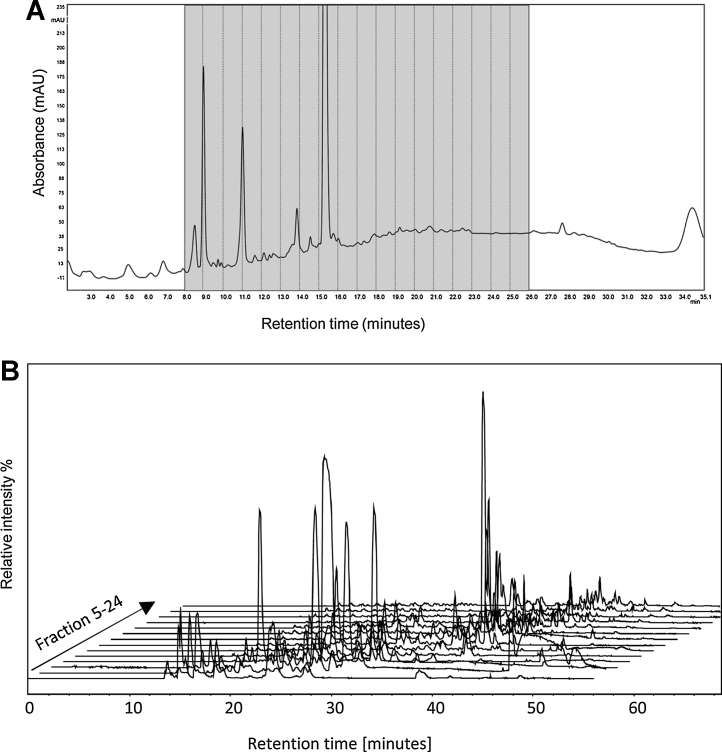
2D-LC analysis of histone peptides from primary monocyte derived macrophages. (A) 1st dimension fractionation of histone peptides using a PGC column. 10 μg of propionylated histone peptides were analysed using a 25 min linear gradient (0–70% Buffer B (97% ACN 0.1% TFA) at a flow rate 0. 25 ml/min. Absorbance at 214 nm. One minute fractions were collected from 8 min to 26 min (highlighted in grey). (B) 2D plot showing the TICs obtained for each 1st dimension fraction. The first dimension fractions were analysed on a C_18_ stationary phase using nano flow chromatography interfaced with mass spectrometry. Peptide separations were performed using Buffer A: 0.1% FA, 3% ACN. Buffer B: 0.1% FA 97% ACN. 3–25% buffer B over 32 min, 25–50% buffer B over 10 min at a flow rate 300 nl/min.

Histone proteins were purified from 14 day old human primary macrophages by acid extraction. 10 μg of histone protein was then subjected to chemical dervitisation by propionic anhydride prior to and post tryptic digestion. The digested histone peptides were subsequently separated and fractionated in the first dimension using a PGC column. Peptide separations were performed under reverse phase conditions (0.1% TFA in conjunction with an increasing acetonitrile gradient, see Fig. 1A). One minute fractions were collected, dried and analysed using nano-flow reverse phase chromatography on a C_18_ stationary phase interfaced to mass spectrometry. When considering fractionation in the first dimension using a 2D-LC approach, a balance must be struck between the length of gradient, number of fractions and downstream MS analysis time. A 2D plot of the base peak chromatogram for each of the second dimension fractions is shown in Fig. 1B. The MS/MS spectra generated were used in conjunction with the peptide retention time profiles to validate the peptide identifications. Relative retention times on C_18_ columns are reliable and well characterised for histone PTMs/peptide proteoforms [14], [33], [34]. Following manual verification of the identified histone PTMs, the PGC column was assessed as a candidate for separation in the 1st dimension using two parameters: orthogonality and fractionation efficiency.

### 3.1 Histone peptide fractionation efficiency

**Fig. 2:**
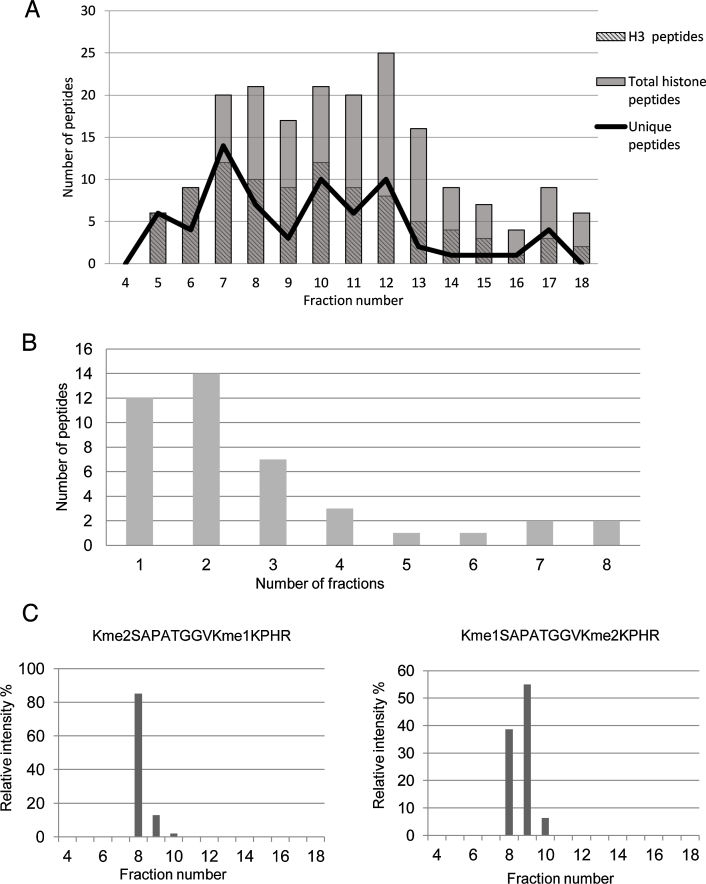
Fractionation of histone peptides on the 1st dimension PGC stationary phase. (A) Fractionation efficiency of the PGC column was assessed by plotting both the number unique and total number of peptides for both all histones and histone H3 that were identified in each fraction. H3 peptides elute first from fraction 5 (12 min) and elute out across the entire gradient. (B) Graph showing the number of fractions the histone peptides elute in the 1st dimension HPLC. (C) Bar chart showing the relative abundance of two histone peptides across multiple fractions. Quantitative analysis of the Kme1STGGKAPR peptide shows 91% elutes in one fraction. 92% of the Kme1SAPATGGVKme3KPHR elutes over 2 fractions.

The fractionation efficiency of the first dimension HPLC separations was evaluated by observing the number of fractions in which each unique peptide was identified [35]. Following 2D-LC MS analysis of the histone peptides, the total number of identified peptides corresponding to histone proteins (H1, H2A, H2B, H3 and H4) in each fraction and those peptides unique to each fraction is shown in Fig. 2A. The results show that the majority of H3 peptides elute in the first 7 fractions. Fraction 12 contains both the highest number of histone peptides and unique peptides (Fig. 2A). The peptide H3K9me3K14ac is the first histone peptide to elute with only a couple of non-histone peptides eluting prior to this. All histone peptides elute within a 16 min window, and it is this window that is used for fractionation in further experiments.

The number of fractions in which the histone peptides eluted is shown in Fig. 2B. Ideally all of a single peptide proteoform will elute in a single fraction, as this reduces sample complexity. The results show that the majority of peptides (75%) elute within 3 fractions or less, with a small amount of peptides eluting out over longer ranges (see Fig. 2B). However, as the fractionation was set for one minute intervals a number of peaks could be distributed across consecutive fractions, despite eluting from the column over a narrow range. Further analysis of the elution reveals that in most cases, >90% of the total peptide elutes in one or two fractions. The relative intensity of two different peptides identified from H3 covering K27-R40 residues (Kme2SAPATGGVKme1KPHR and Kme1SAPATGGVKme2KPHR) across the different fractions is shown in Fig. 2C. The results show that the majority of these peptides elute over one or two fractions. Peptides that elute over a longer range are retained more strongly by the PGC column. It is important to note that the use of fractionation of the sample prior to MS analysis does not significantly alter the accuracy of peptide quantification. A comparative quantitative analysis of PTMs from a number of histone peptides from both 1D and 2D LC–MS is shown in supplementary Fig. 2.

### 3.2 Orthogonality of 2D peptide separations

**Fig. 3:**
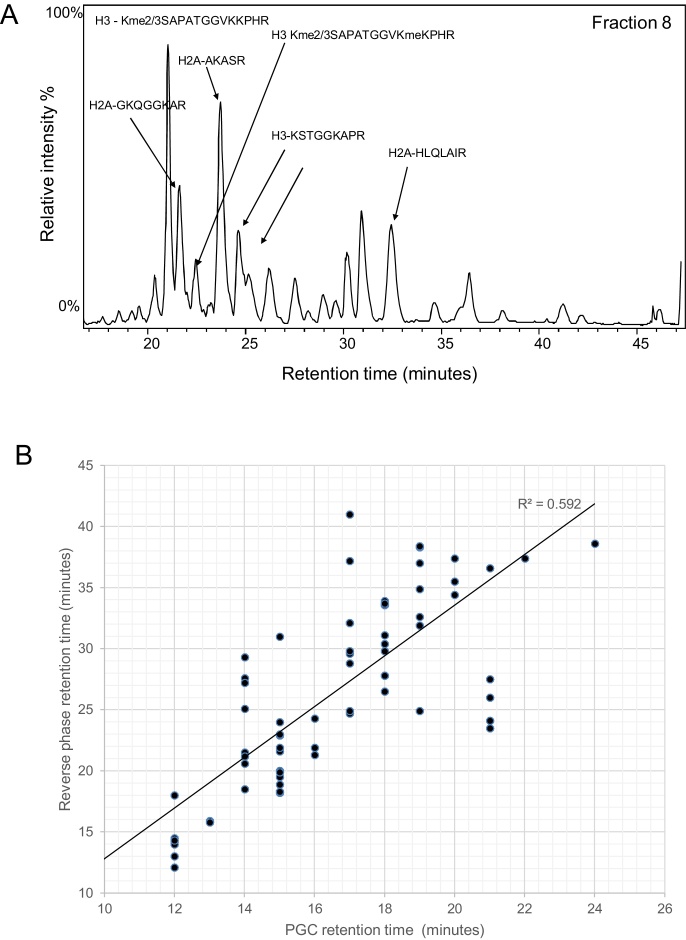
Orthogonality of histone peptide separations using 2D-LC. (A) Orthogonality is exemplified by the BPC trace of a single fraction (8) from the PGC 1st dimension analysis. A number of identified histone peptides are highlighted across the 2nd dimension. (B) 2D plot comparing the retention time of all identified histone peptides in both the 1st and 2nd dimension. Each dot represents a single peptide, with only the fraction where the species is most abundant is shown.

Histone peptides represent a good test of the orthogonality between PGC and conventional C_18_ stationary phases because Arg-C like tryptic peptides have a wide range of hydrophobicities, including small hydrophilic peptides, for example H3K4 peptides that are not well retained on C_18_ media. In order to assess orthogonality, the base peak chromatogram (BPC) of each 1-min fraction was analysed. The 2D-LC plot of the BPC for each of the fractions from the 1st dimension demonstrates the orthogonality of the two different phases (see Fig. 1B). Further analysis of fraction 8 is shown in Fig. 3A. The LC-MS results show a number of different peptides are present in this single 1 min fraction which are subsequently separated and identified across the 2nd dimension. The corresponding abundant peptides identified by mass spectrometry in this fraction are highlighted (see Fig. 3A). Further analysis comparing the retention times of all identified histone peptides is shown in Fig. 3B. The results show the distribution of retention times of the identified peptides in the first and second dimensions. The results demonstrate the orthogonality between the two dimensions for the analysis of histone peptides, consistent with previous shotgun proteomic analysis [31].

### 3.3 Elution profiles of modified histone peptides

**Fig. 4:**
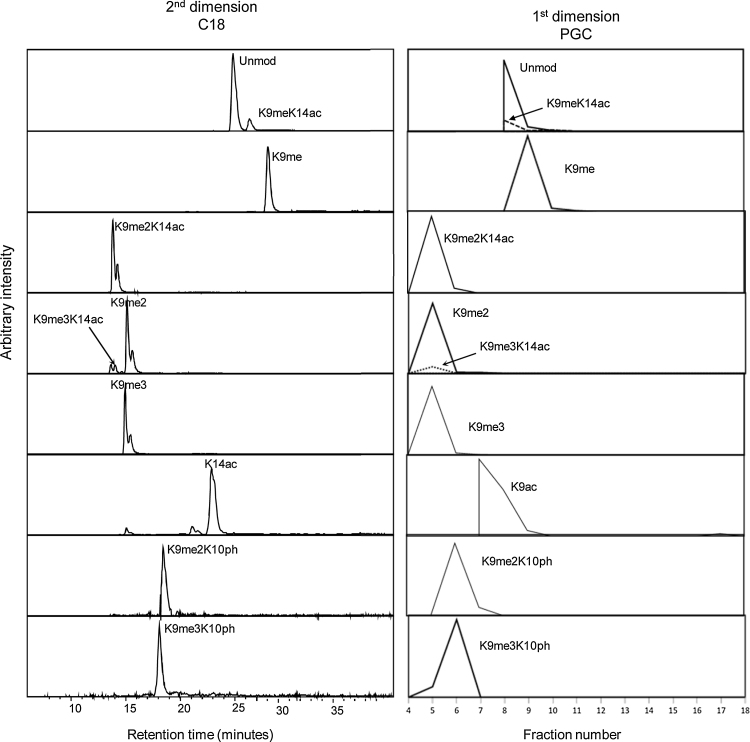
A comparison of the elution profiles of H3 K9-17 proteoforms on the C_18_ and PGC stationary phases. (A) EICs were generated for all proteoforms of peptide K9-17 [M+2H]^2+^ from the C_18_ second dimension analysis. (B) Total area of the EICs for all proteoforms of peptide K9-17 [M+2H]^2+^ plotted against fraction number in the PGC first dimension.

Both the LC–MS retention times and elution profiles of the histone peptides offer useful additional information for the identification/verification of histone post translational modifications. A defined elution profile on a C_18_ column has been well characterised for a number of peptides and their modified forms [14], [24]. In order to assess the elution profiles on the PGC column under the reverse phase conditions employed in this study, the elution profile of the H3 K9-14 peptide KSTGGKAPR was further analysed. The elution profile of the modified peptides on the C_18_ column compared to the fraction of elution plotted against the intensity in the first dimension is shown in Fig. 4. A similar elution profile can clearly be seen on the PGC in comparison with the C_18_ stationary phase. The K9me species is the latest eluting peptide, with the K9me2 and K9me3 and dual modification versions K9me2/3K14ac eluting first. There is greater overlap in elution times on the PGC stationary phase compared to the C_18_ column, probably as a result of the reduced resolution when viewing the first dimension compared to the second. The H3K9me3 and H3K9/14ac which vary in mass by only 0.036 Da have distinctly different retention times [24]. In the 2D-LC analysis, the two peptides elute in separate fractions on the PGC 1st dimension (see Fig. 4). This therefore represents an additional level of confirmation, without requiring high mass resolution mass spectrometry analysis to discriminate between the two species.

### 3.4 A comparative study between offline 2D-LC and 1D-LC analysis of histone PTMs

Following assessment of the 2D-LC MS method, a comparative study between the offline 2D-LC, and two alternative 1D-LC methods was performed. 1D-LC methods using offline desalting via PGC tips and online desalting via C_18_ were performed to compare the number of histone post translational modifications identified and quantified using each approach. All the studies were combined with a nano-flow C_18_ stationary phase in conjunction with reverse phase chromatography interfaced with the maXis UHR TOF mass spectrometer.

**Fig. 5:**
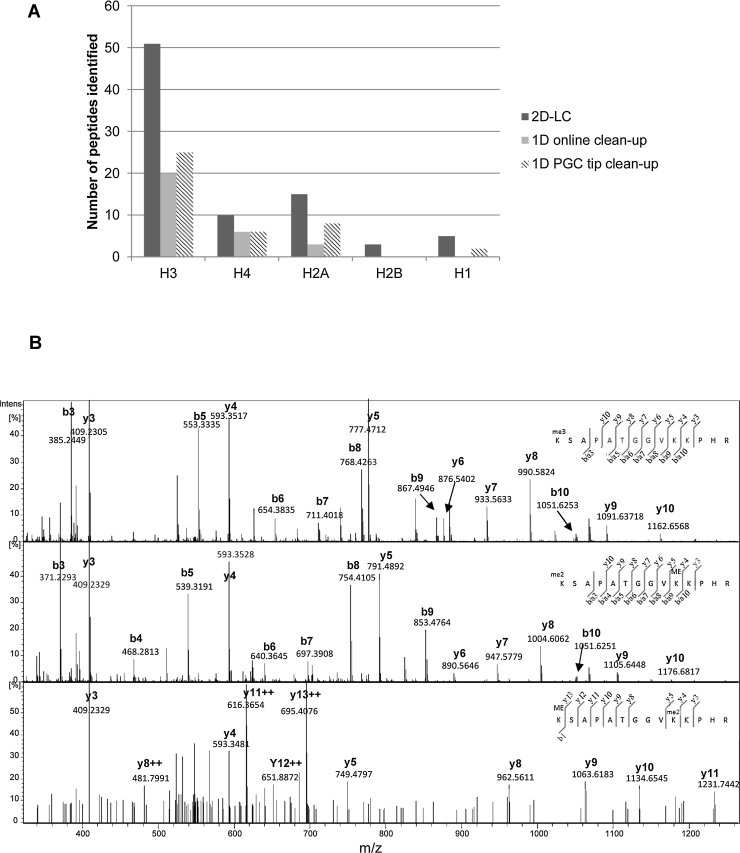
Comparative analysis of histone PTMs from MDMs using 1D and 2D-LC–MS. (A) Bar chart summarising the number of histone peptides and PTMs identified. Three different bottom-up methodologies were compared based on the number of histone peptides and PTMs identified. The methods compared are offline 2D-LC using a PGC column in the 1st dimension, 1D-LC method using an offline PGC tip desalting and a 1D-LC approach using online desalting (C_18_). In all cases a total of 5 μg of digested histone protein was used for LC–MS/MS analysis. (B) MS/MS spectra of a number of modifications identified on H3. The prominent y and b ions are highlighted. The 2D-LC–MS identified all three peptides shown. In contrast, 1D-LC MS using offline desalting identified K27me3 and K27me2K36me and 1D-LC MS online desalting only identified K27me3.

10 μg of histone protein extracted from primary monocyte derived macrophages were chemically derivatised with propionic anhydride prior to and post tryptic digestion. A total of 14 fractions from the 2D-LC MS compared with 1D-LC MS methods. The results are summarised in Fig. 5 and Supplementary Table 1. Focusing on histone H3, the best characterised histone protein, 2D-LC analysis enabled the identification of 51 histone peptides. In contrast, the 1D-LC online or offline desalting identified 20 and 25 histone peptides respectively (see Fig. 5A). Similarly the number of modifiable sites identified was increased in the 2D-LC method with 11 modifiable sites identified, compared to 9 for the 1D-LC online or offline desalting methodologies. The same trend can be seen with histone H2A, H2B, H4 and H1. The number of peptides identified using the 1D-LC methods using offline desalting on porous graphitic solid phase extraction tips is similar to the number found previously using the same technique [24]. Overall, fewer peptides were detected using online desalting compared to the offline desalting on the PGC tip. No peptides were detected by the 1D-LC methods that were not detected by the 2D-LC approach. Peptides that were not detected in the online C_18_ desalt method are those that are the most hydrophilic (K3-8 and K9-17) peptides. These small, hydrophilic peptides are poorly retained on C_18_ columns even in the presence of 0.1% TFA as the ion pair reagent and it is likely that they desorb from the C_18_ surface during the wash step. In addition, the 2D-LC method enabled the identification of a number of low abundant histone modifications in contrast to the 1D-LC methods. This may be in part due to the increase in dynamic range that is afforded by using an offline separation with a PGC column upstream of LC–MS [32]. The MS/MS spectra of a number of modified peptides identified across the different methods is shown in Fig. 5B. All three peptides (H3K27me3, K27me2K36me and K27meK36me2) were identified using the 2D-LCMS. In contrast, 1D-LC MS in conjunction with offline desalting was unable to identify K27meK36me2 and 1D-LC MS in conjunction with online desalting only identified K27me3 peptide. All histone peptides identified in this study for each of the methodologies is summarised in Supplementary Tables 1 and 2.

The 2D-LC MS approach allowed for the highest number of histone PTMs to be consistently identified across each of the LC MS methods. The ability to detect the maximum number of possible proteoforms for each peptide is of particular importance because the histone PTMs on each peptide are expressed as a relative percentage. Thus the absence of modified peptides can cause the data to become skewed and therefore not representative of the true histone landscape. Furthermore, with the application of correction factors to normalise the inherent effects of modifications on peptide ionisation efficiency, the ability to reliably detect as many possible proteoforms for each peptide is important for accurate relative quantification [11].

The number of H3 histone peptide proteoforms identified using the PGC-based 2D-LC analysis in this study was 51, which is an improvement on the 22 detected in a previous SCX-based analysis on a different cell type [30]. Other studies using a wide range of methodologies both with 2 dimensional separations and without (1D-LC) have found similar amounts of histone PTMs, including many PTMs that were not identified in this study [11], [29], [36], [37], [38], [39]. However, comparative analysis between different studies is challenging, as different cell types or often different organisms are used, which have different histone PTM profiles and so caution should be applied when comparing two different methods. For example, H3K36me2 was not detected in MDMs, but previous research has detected this modification in *Apis melifera* [40] using a 1D-LC methodology. Moreover, the mass spectrometer and method of data acquisition (e.g. DDA vs SWATH) used will have significant impact on histone PTM detection. Mass spectrometers have different capabilities in terms of sensitivity, dynamic range and resolution, all of which affect the accurate identification and quantification of histone PTMs. Finally, the search algorithm used can also influence the number of histone PTMs identified [41].

### 3.5 High throughput quantification of histone PTMs using a bioinformatics pipeline

The major benefit of the 2D-LC set-up is that it allows the increased identification and quantification of histone PTMs compared to 1D-LC–MS approaches. However, the trade-off is the increase in both MS and downstream data processing time. In order to improve the data processing a script was developed in the Visual Basic language (VB). The workflow of the downstream processing pipeline that was developed, termed Histomatic, is shown in Supplementary Fig. 3.

Following the creation of the .mgf file by Data Anlaysis v.4.1 (DA) and database search in MASCOT all identified peptides were manually verified by first generating the extracted ion chromatogram (EIC) for the specified peptide and correlating this with the identified peptide in MASCOT. Subsequently the parent ion is first checked to be in the correct charge state and the MS/MS spectra is verified against the theoretical MS/MS spectra. The time at the apex of the EIC peak for which the peptide was identified is recorded and placed into Histomatic. The script generates peptide EICs for each fraction then carries out peak assignment, smoothing and integration. Histomatic exports these values and correction factors can be applied to determine the relative abundance of each peptide proteoform (see Supplementary Fig. 3).

### 3.6 Identification and quantification of histone PTMs in primary monocyte derived macrophages (MDMs)

**Fig. 6:**
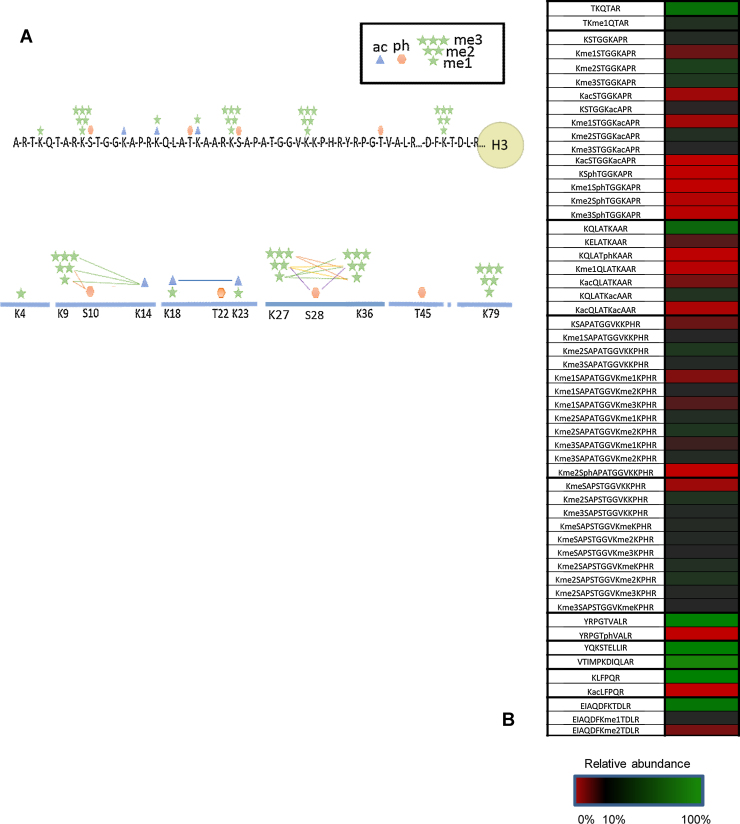
Identification and quantification of histones H3 PTMs and their combinatorial patterns in primary monocyte derived macrophages. (A) Schematic illustration summarising the modifications identified and the combinatorial nature of these modifications on histone H3 using mass spectrometry analysis. The modifications identified were focused on the N-terminal tails in both histone proteins. Lysine residues were the main site of modification. Mono, di and trimethylation (me1, me2 and m3) and acetylation (ac) were the most abundant modifications identified with lower levels of phosphorylation (ph) identified. B) A heat map depicting the relative abundance of all human primary MDM histone H3 peptides identified. Red = 0%, Green = 100%. (For interpretation of the references to colour in this figure legend, the reader is referred to the web version of this article.)

Using 2D-LC MS analysis in conjunction with the in-house Histomatic script for peptide quantification, a total of 51 H3 peptides, 10 H4 peptides, 15 H2A peptides, 3 H2B peptides and 5 H1 histone peptides were identified and quantified from primary monocyte derived macrophages. All modified peptides identified were manually validated with respect to both position and type of modification. All resulting MS data and MS/MS spectra are shown in Supplementary Table 2 and Supplementary Fig. 4. Short range combinations of histone PTMs were also detected on the same tryptic peptide, the most complex of these patterns was found on histone H3 (see Fig. 6A). In total, we were able to detect 61 specific modification states on 12 distinct peptides of H3 and H4. The relative abundance of all modified peptides from histone proteins from primary monocyte derived macrophages is shown in Supplementary Fig. 5. Fig. 6B summarises the histone H3 PTMs in primary monocyte derived macrophages (MDMs) and includes the relative abundance of the different H3 modified peptides identified in this study.

The implementation of the 2D-LC methodology enabled increased numbers of histone PTMs to be identified and quantified in an unbiased manner compared to 1D-LC methodologies. The ability to detect and quantify such a wide range of histone PTMs in primary cells is advantageous compared to in-vitro cell based models as they will more closely reflect the *in-vivo* response. In addition, the use of mass spectrometry allows the local context of the histone PTMs to be understood, which is vital in determining their biological function.

## 4 Conclusions

Epigenetic modifications are increasingly recognized as playing an important role in the pathogenesis of infectious diseases. They represent a critical mechanism regulating transcriptional profiles in the immune system that contribute to the cell type and stimulus specificity of the transcriptional response. Therefore analysing changes in histone PTMs is important to study the role of epigenetics in the pathogenesis of infectious diseases. The offline HPLC fractionation of peptides generated from the bottom up analysis of acid-extracted histone proteins from primary monocyte derived macrophages was performed using a PGC column in the first dimension, in conjunction with LC–MS analysis using a conventional C_18_ stationary phase in the second dimension. This first dimension separation uses typical reverse phase solvents that are readily compatible with the second dimension following acetonitrile removal and shows a high degree of orthogonality to C_18_ RP separations. Moreover, in contrast to SCX-based 2D-LC methods to fractionate histone peptides, no desalting or buffer exchange of the sample is required prior to LC–MS analysis. Using this approach a 51% increase in peptides were identified and quantified compared to traditional 1D-LC analysis on the same MS instrument. This method enabled lower abundance histone peptides to be identified and reliably quantified. We were able to identify 51 H3 peptides from primary monocyte derived macrophages. In addition, a number of peptides were generated that contained multiple PTM sites thus enabling the elucidation of combinatorial patterns. The in house data processing pipeline developed in this study enables the rapid, automated and high throughput processing of data generated from 2D-LC–MS/MS experiments for the identification and quantification of histone PTMs.
