# Supplementary material for: Analysis of histone post translational modifications in primary monocyte derived macrophages using reverse phase × reverse phase chromatography in conjunction with porous graphitic carbon stationary phase
Source: J Chromatogr A. 2016 Jul 1;1453:43–53. doi: 10.1016/j.chroma.2016.05.025 (PMC4906248; doi:10.1016/j.chroma.2016.05.025)
Supplement: Supplementary file 1 [file mmc1.docx]

***Supplementary information***

| 2D-LC | 1D-LC (offline desalt) | 1D-LC (online desalt) |
| --- | --- | --- |
| TKQTAR | TKQTAR | KSTGGKAPR |
| TKme1QTAR | TKme1QTAR | Kme1STGGKAPR |
| KSTGGKAPR | KSTGGKAPR | Kme2STGGKAPR |
| Kme1STGGKAPR | Kme1STGGKAPR | Kme3STGGKAPR |
| Kme2STGGKAPR | Kme2STGGKAPR | KacSTGGKAPR |
| Kme3STGGKAPR | Kme3STGGKAPR | KSTGGKacAPR |
| KacSTGGKAPR | KacSTGGKAPR | Kme1STGGKacAPR |
| KSTGGKacAPR | KSTGGKacAPR | Kme2STGGKacAPR |
| Kme1STGGKacAPR | Kme1STGGKacAPR | Kme3STGGKacAPR |
| Kme2STGGKacAPR | Kme2STGGKacAPR | KQLATKAAR |
| Kme3STGGKacAPR | Kme3STGGKacAPR | Kme1QLATKAAR |
| KacSTGGKacAPR | KQLATKAAR | KacQLATKAAR |
| KSphTGGKAPR | Kme1QLATKAAR | KQLATKacAAR |
| Kme2SphTGGKAPR | KacQLATKAAR | KacQLATKacAAR |
| KQLATKAAR | KQLATKacAAR | Kme2SAPATGGVKKPHR |
| KQLATphKAAR | KacQLATKacAAR | Kme3SAPATGGVKKPHR |
| Kme1QLATKAAR | KSAPATGGVKKPHR | Kme2SAPATGGVKme1KPHR |
| KacQLATKAAR | Kme2SAPATGGVKKPHR | Kme2SAPATGGVKme2KPHR |
| KQLATKacAAR | Kme3SAPATGGVKKPHR | YRPGTVALR |
| KacQLATKacAAR | Kme2SAPATGGVKme1KPHR | KLFPQR |
| KSAPATGGVKKPHR | Kme2SAPATGGVKme2KPHR | GKGGKGLGKGGAKR |
| Kme1SAPATGGVKKPHR | Kme3SAPATGGVKme1KPHR | GKGGKGLGKGGAKacR |
| Kme2SAPATGGVKKPHR | YRPGTVALR | GKGGKacGLGKGGAKacR |
| Kme3SAPATGGVKKPHR | YQKSTELLIR | GKGGKacGLGKacGGAKR |
| Kme1SAPATGGVKme1KPHR | EIAQDFKTDLR | DNIQGITKPAIR |
| Kme1SAPATGGVKme2KPHR | GKGGKGLGKGGAKR | GVLKVFLENVIR |
| Kme1SAPATGGVKme3KPHR | GKGGKGLGKGGAKacR | AKAKTR |
| Kme2SAPATGGVKme1KPHR | GKGGKacGLGKGGAKacR | HLQLAIR |
| Kme2SAPATGGVKme2KPHR | GKGGKGLGKacGGAKacR | KGHYAER |
| Kme2SAPATGGVKme3KPHR | GKGGKacGLGKacGGAKR | AGLQFPVGR |
| Kme3SAPATGGVKme1KPHR | DNIQGITKPAIR |  |
| KSphAPATGGVKKPHR | AKAKSR |  |
| Kme2SphAPATGGVKKPHR | AKAKTR |  |
| YRPGTVALR | HLQLAIR |  |
| YQKSTELLIR | DNKKTR |  |
| YEKSTELLIR | GKTGGKAR |  |
| KLFPQR | KGHYAER |  |
| EIAQDFKTDLR | GKacQGGKAR |  |
| EIAQDFKme1TDLR | AGLQFPVGR |  |
| EIAQDFKme2TDLR | GKQGGKAR |  |
| KSAPSTGGVKKPHR | KSAGAAKR |  |
| Kme1APSTGGVKKPHR | KmeSAGAAKR |  |
| Kme2SAPSTGGVKKPHR |  |  |
| Kme3SAPSTGGVKKPHR |  |  |
| Kme1SAPSTGGVKme1KPHR |  |  |
| Kme1SAPSTGGVKme2KPHR |  |  |
| Kme1SAPSTGGVKme3KPHR |  |  |
| Kme2SAPSTGGVKme1KPHR |  |  |
| Kme2SAPSTGGVKme2KPHR |  |  |
| Kme2SAPSTGGVKme3KPHR |  |  |
| Kme3SAPSTGGVKme1KPHR |  |  |
| GKGGKGLGKGGAKR |  |  |
| GKGGKacGLGKGGAKR |  |  |
| GKGGKGLGKacGGAKR |  |  |
| GKGGKGLGKGGAKacR |  |  |
| GKGGKacGLGKGGAKacR |  |  |
| GKGGKGLGKacGGAKacR |  |  |
| GKacGGKacGLGKacGGAKacR |  |  |
| Kme2VLRDNIQGITKPAIR |  |  |
| DNIQGITKPAIR |  |  |
| DNIQGITphKPAIR |  |  |
| DAVTYTEHAKR |  |  |
| AKAKSR |  |  |
| AKAKTR |  |  |
| HLQLAIR |  |  |
| DNKKTR |  |  |
| GKTGGKAR |  |  |
| KGHYAER |  |  |
| GKmeQGGKAR |  |  |
| GKacQGGKAR |  |  |
| AGLQFPVGR |  |  |
| NDEELNKLLGR |  |  |
| AGGKAGKDSGKAKTKAVSR |  |  |
| AGGKAGKDSGKAKAKAVSR |  |  |
| GKQGGKAR |  |  |
| HLQLAVR |  |  |
| GGKKKSTKTSR |  |  |
| KSAGAAKR |  |  |
| KmeSAGAAKR |  |  |
| SETAPAAPAAPAPAEKTPVKKKAR |  |  |
| SETAPAETATPAPVEKSPAKKKATKKAAGAGAAKR |  |  |
| GAPAAATAPAPTAHKAKKAAPGAAGSR |  |  |
| STITSR |  |  |
| EIQTAVR |  |  |
| LAHYNKR |  |  |
|  |  |  |
|  |  |  |
|  |  |  |

**Supplementary Table 1. Corresponding peptides identified by 2D-LC and 1D-LC methods.** Histone peptides identified in each of the three methods from monocyte derived macrophages. The coloured background represents a different histone protein. Blue = H3.1/ H3.2 peptides, yellow= H3.3 peptides, orange H4=peptides, purple= H2A peptides, grey = H1 peptides, green=H2B peptides.


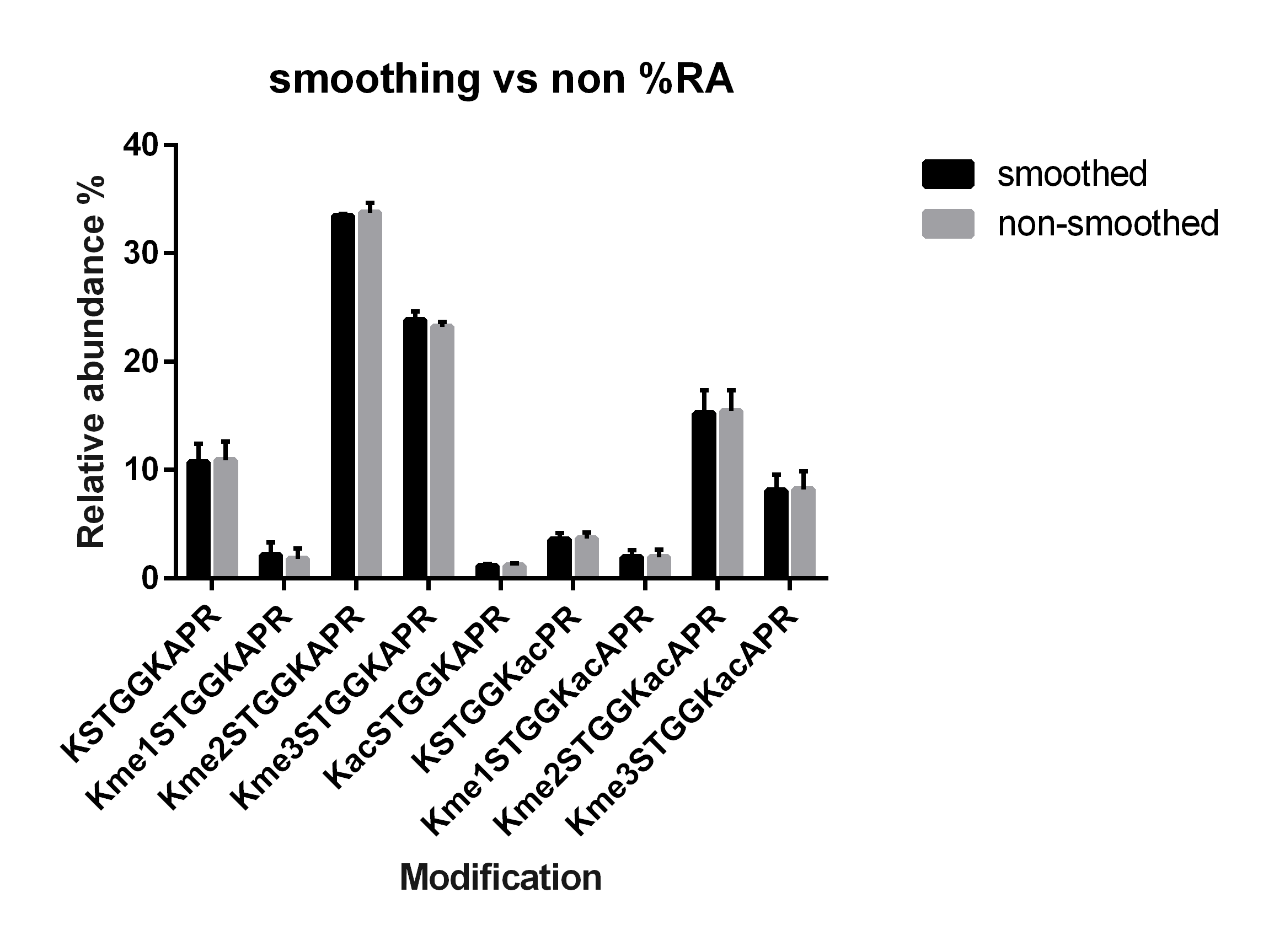


**MDM H3K9-17**

**Supplementary Figure 1. The impact of using Gaussian smoothing on relative quantification of histone PTMs .** A comparison of the effect of using a Gaussian algorithm to smooth extracted ion chromatograms used for the relative quantification of the peptide H3K9-17. The same monocyte derived macrophage samples were used in the comparison with an n=3. All data is shown as mean with SEM. All data plotted and statistics calculated using GraphPad Prism. No significant differences in the relative abundance were introduced by the use of the Gaussian algorithm.

B)

A)


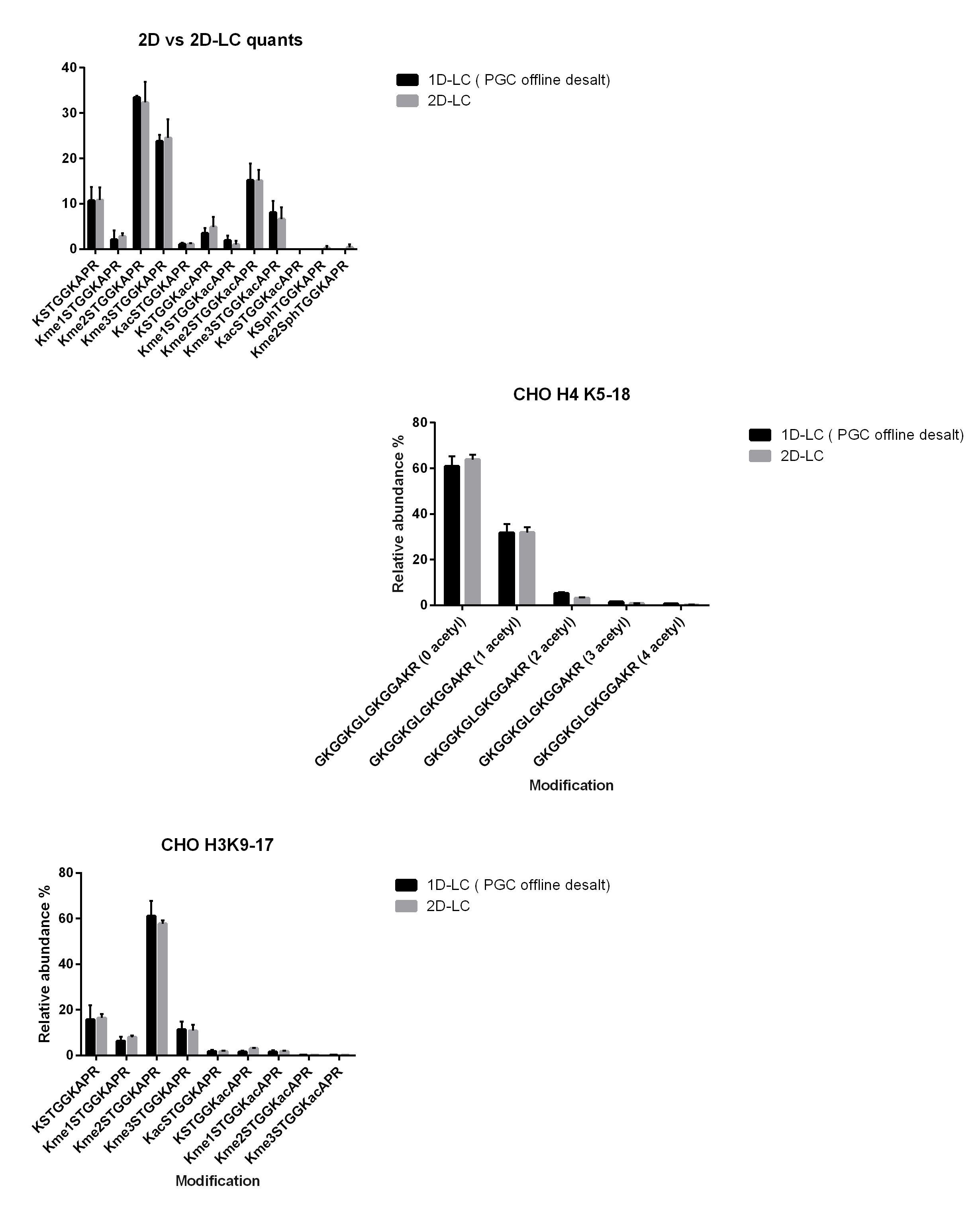

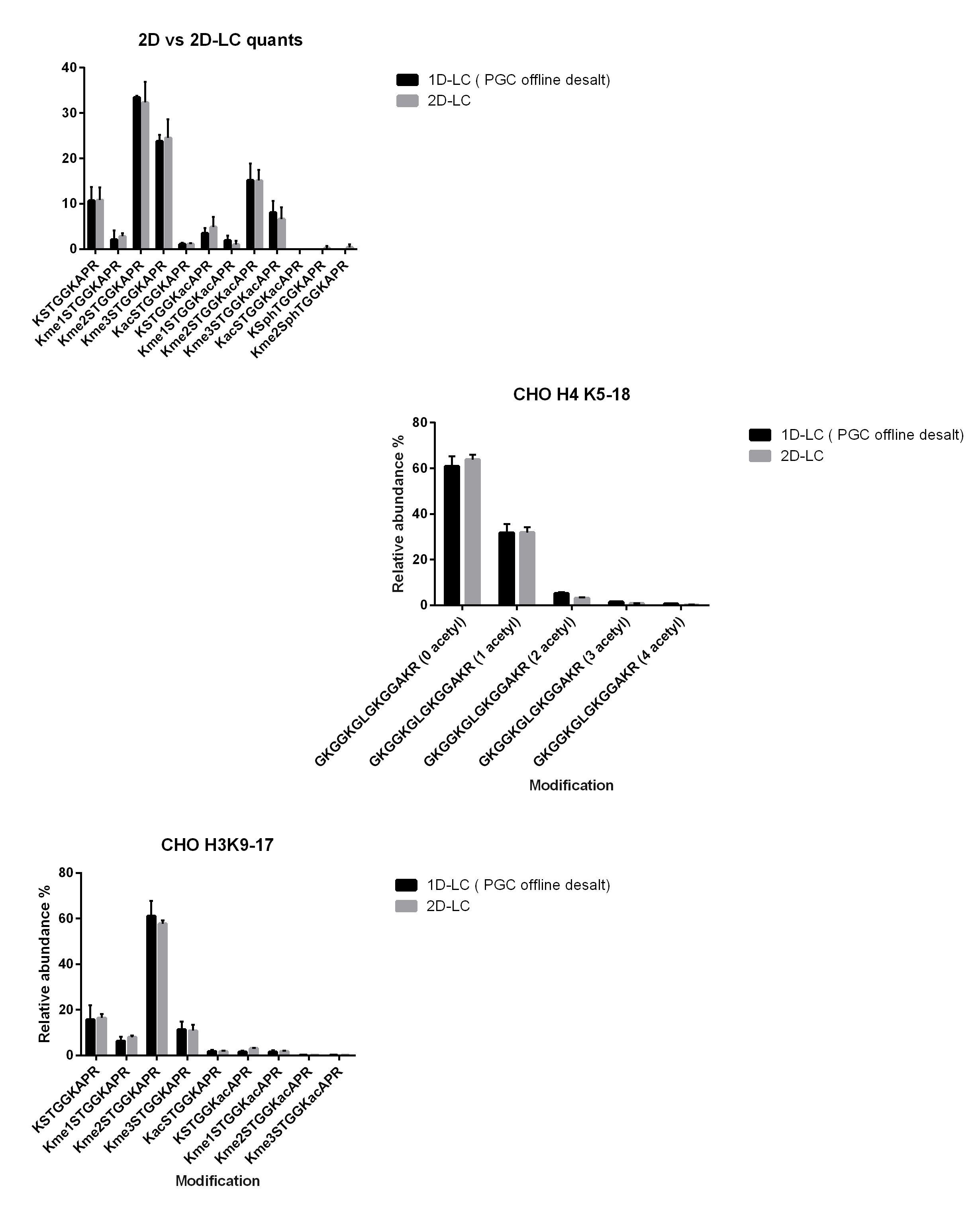


**MDM H3K9-17**


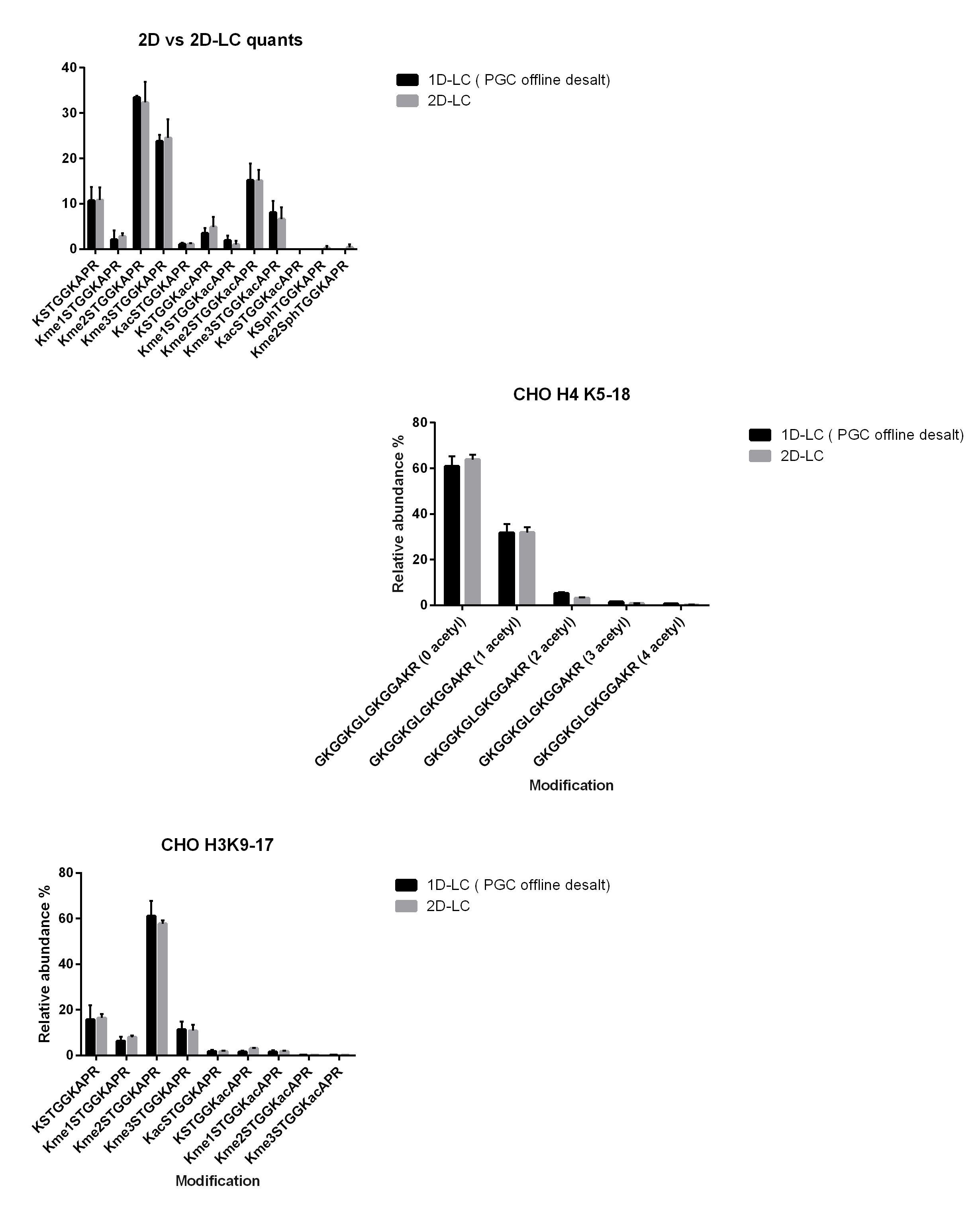


C)

**Supplementary Figure 2. The impact of 2D fractionation on peptide quantification.**

A comparison of the effect of the summation of EIC over multiple limited fractions on the relative abundance compared to a 1D “one-pot” approach. A) histone H4 peptide K5-18 B) Histone H3 K9-17 both from Chinese hamster ovary (CHO) cells. C) histone H3 peptide K9-17 from monocyte derived macrophages. H4 peptide CHO both conditions n=4, H3 peptide CHO both conditions n=3 and H3 peptide MDM 2D-LC n=4, 1D-LC (tip) n=3. All data is shown as mean with SEM. All data plotted and statistics calculated using GraphPad Prism. No significant differences in relative abundance can be seen between the two methodologies. This is consistent in different cell types and peptide


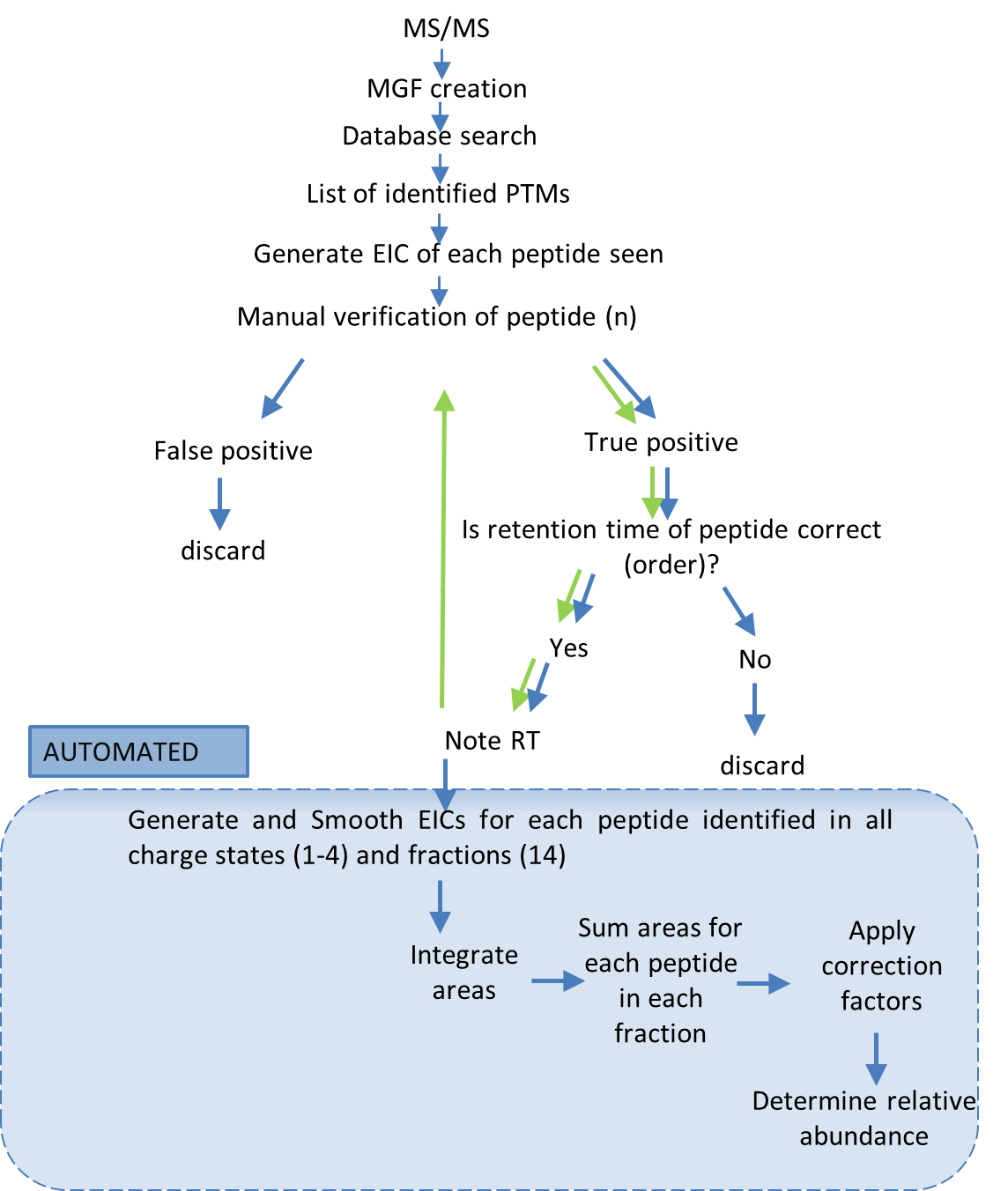


**Supplementary Figure 3. Workflow summarising the data analysis pipeline for the identification and quantification of histone peptides.**

Following the acquisition of mass spectrometry data, an .mgf is created using Data Analysis software (Bruker), this file is then uploaded into the MASCOT database, and used alongside raw data, to manually verify the peptide identification. During this process, the retention time (RT) of the peaks containing the peptide hits that have passed manual verification is recorded and assessed as additional evidence. The RTs are then entered into the Histomatic which allows the automation of the integration and recording of peak areas used in quantification for all fractions.

**Histomatic (additional information)**

Following the creation of the .mgf file by DA and database search in MASCOT all identified peptides were manually verified by first generating the extracted ion chromatogram (XIC/EIC) for the specified peptide, and correlating with the identified peptide in MASCOT. Subsequently the parent ion is first checked to be in the correct charge state and if this is correct, then the MS/MS spectra is verified against the theoretical MS/MS spectra generated by MASCOT. The time at the apex of the EIC peak for which the peptide has been identified, is then recorded. This process is repeated until all peptides have been manually verified. This information is then placed into the Histomatic (If multiple elution times are observed for example when double peaks are present in the EIC both of which contain the identified peptide, then both RT are added).

- The Histomatic will then generate the specified EICs in DA with a range of +/- 0.025 m/z for every entered peptide that has a RT of >0. Unless, the EIC has been added previously i.e. for isobaric PTMs such as H3K27meK36me2 & H3K27me2K36me by using an array function.
- The Histomatic then smooths all the generated EICs and integrates them, generating a compound list.
- Following integration, the DA file is saved in its current state and the compound list is exported into a .csv file and saved into a folder on the C-drive known as ZZHistomatic (which must be made by user).
- The Histomatic, then uses this file to search for the EIC that corresponds with the RT entered (within an error range of +/- 0.2 min) to allow for retention time variation between fraction runs.
- Once these two variables are paired up, the line from the exported compound list, including Area, S/N, range etc. is pulled out and inputted into the Histomatic table
  - In the case of there being two RT, separated by a slash, the sum of the area, S/N and intensity values are displayed.
  - The range values are listed as two separate values separated by a comma

**Supplementary Figure 4**

**Macrophage Histone PTMs MASCOT H3, H4, H2A, H2B, H1**

The following section displays the Mascot output for all identified and manually verified histone PTMs

**H2A, H2B and H1 peptides**

**
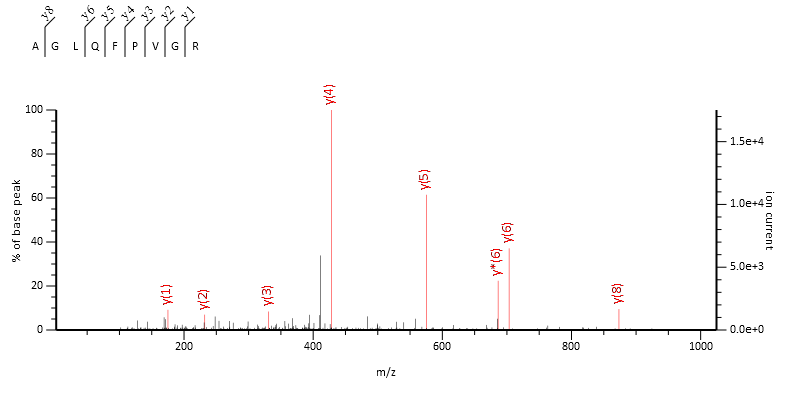

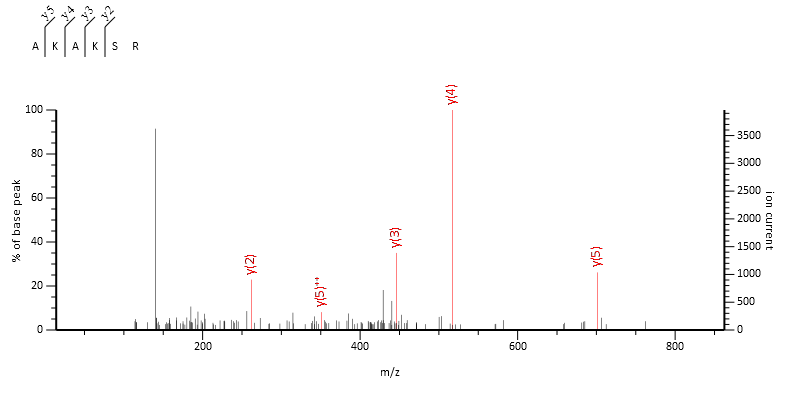

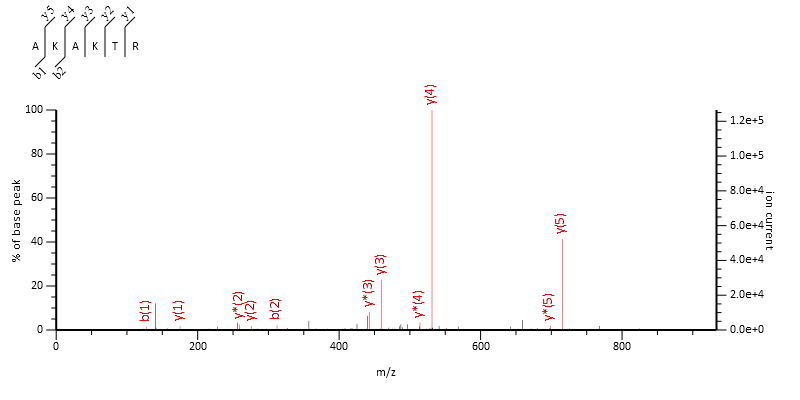

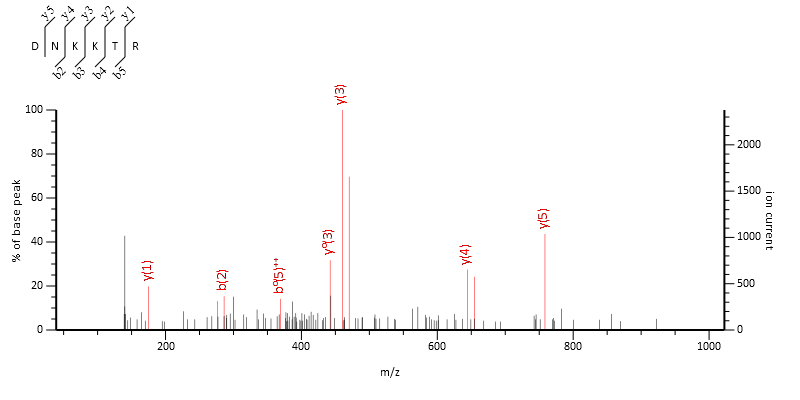

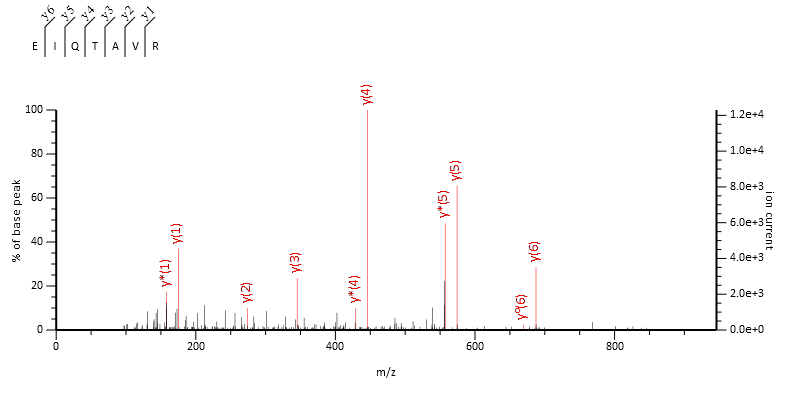

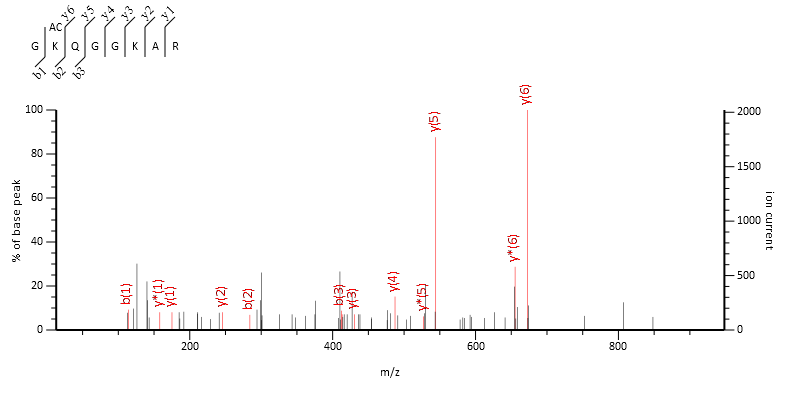

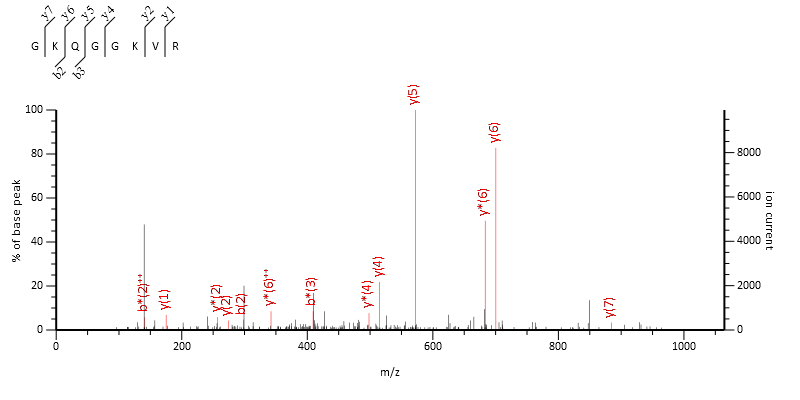

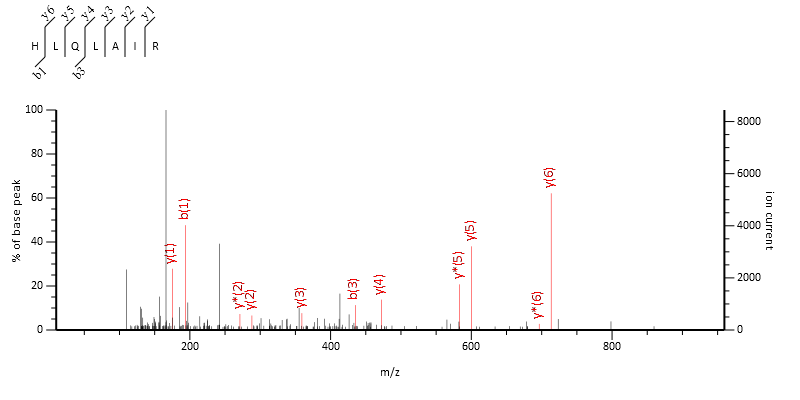

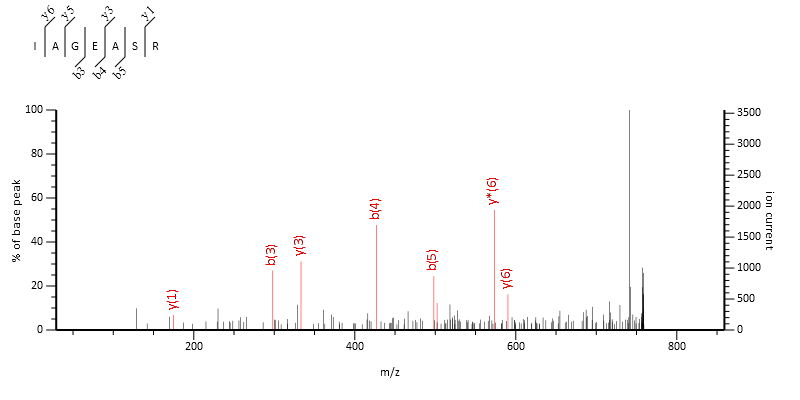

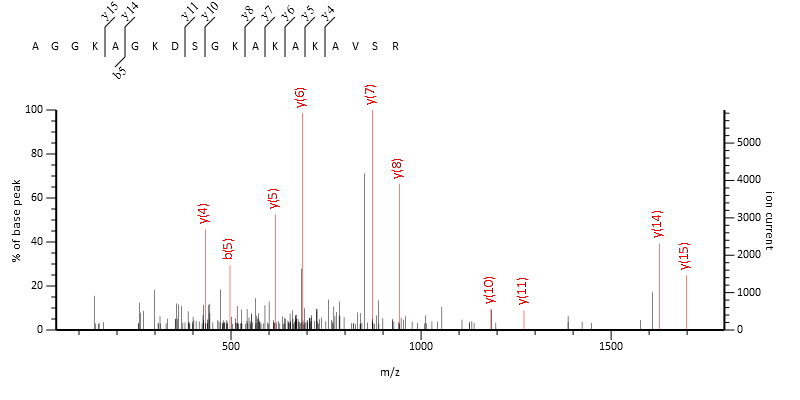

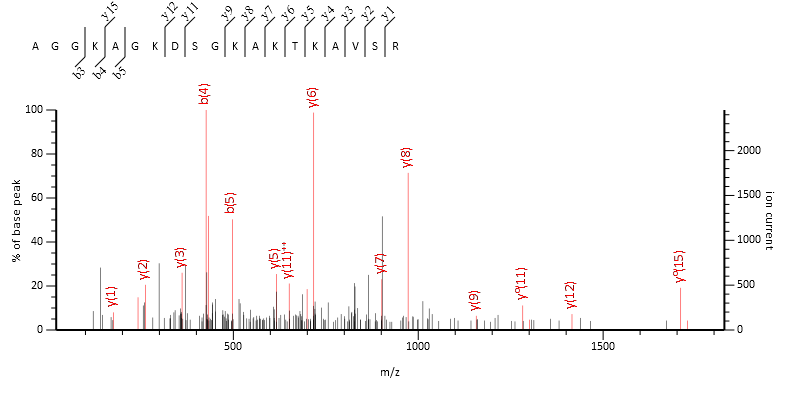

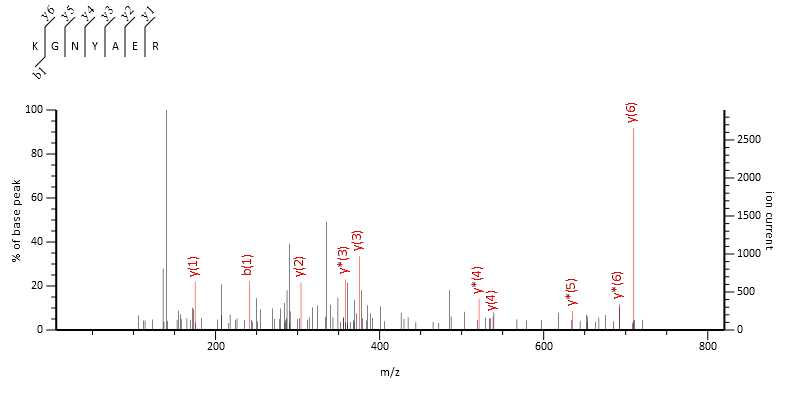

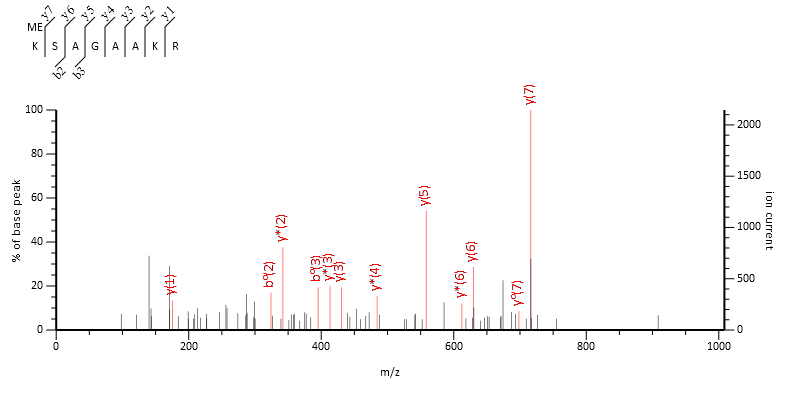

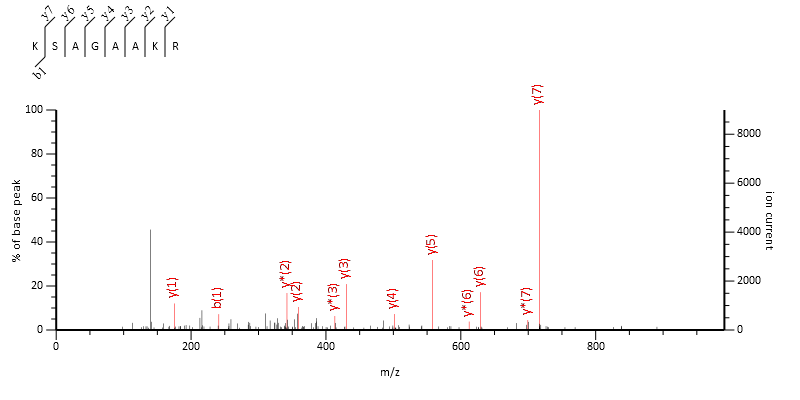

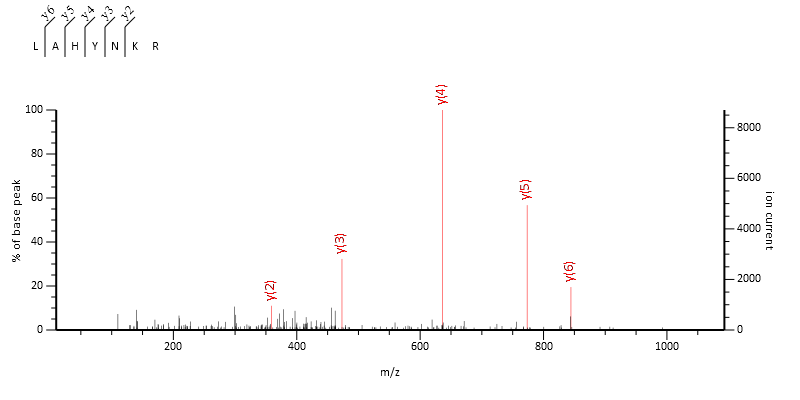

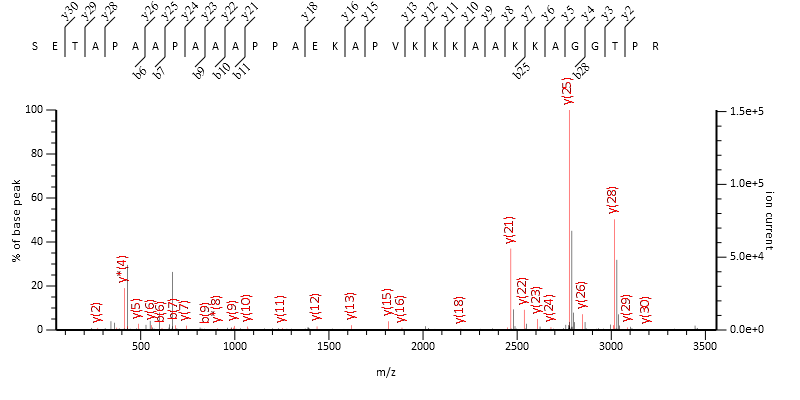

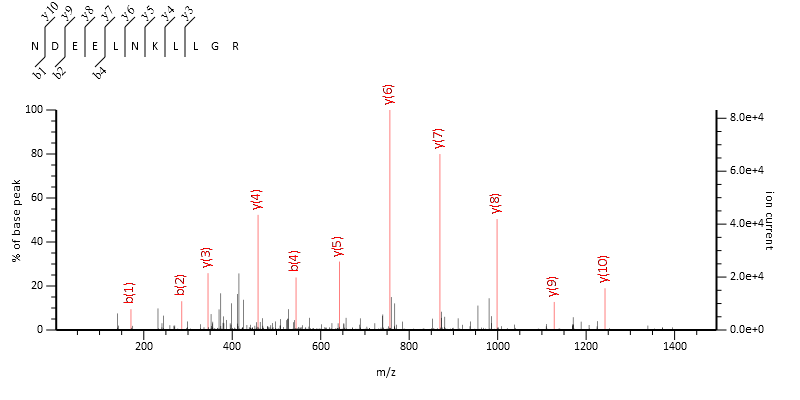
**

**Histone H3.1/2 and H3.3 peptides**

**
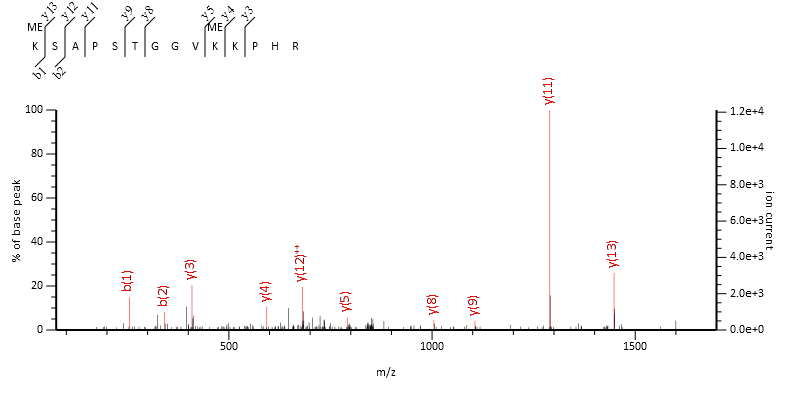

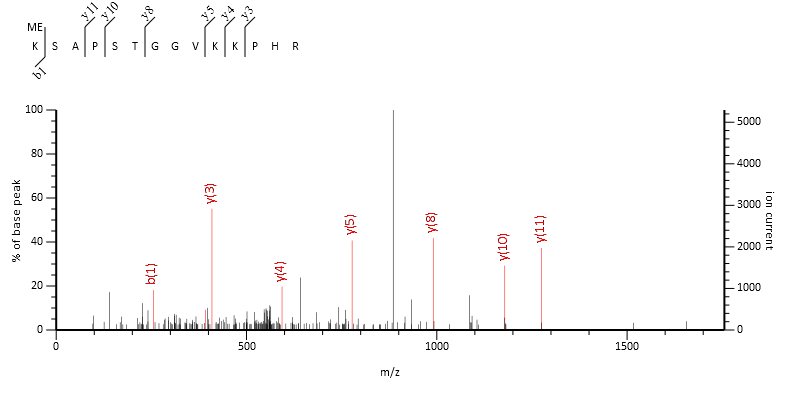

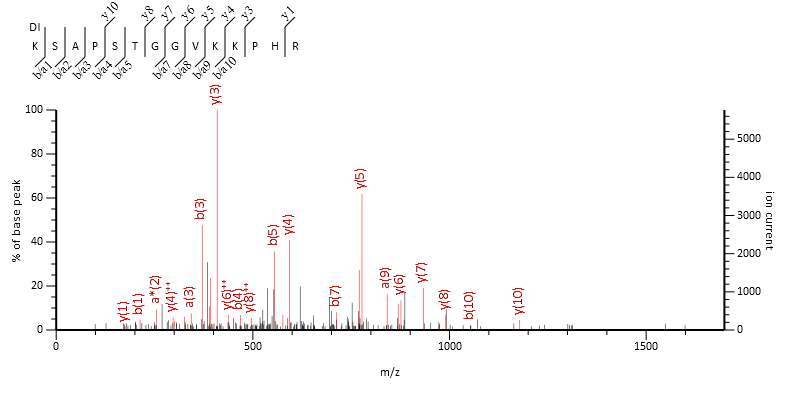

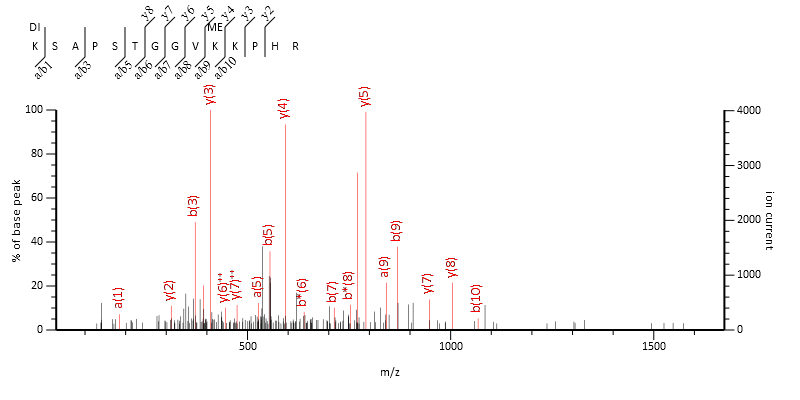

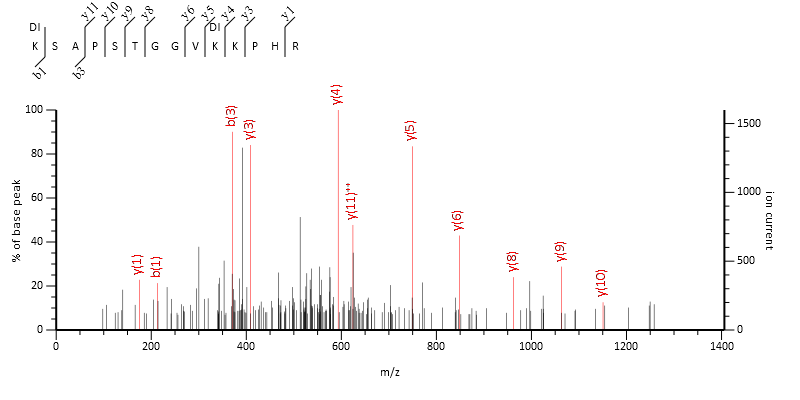

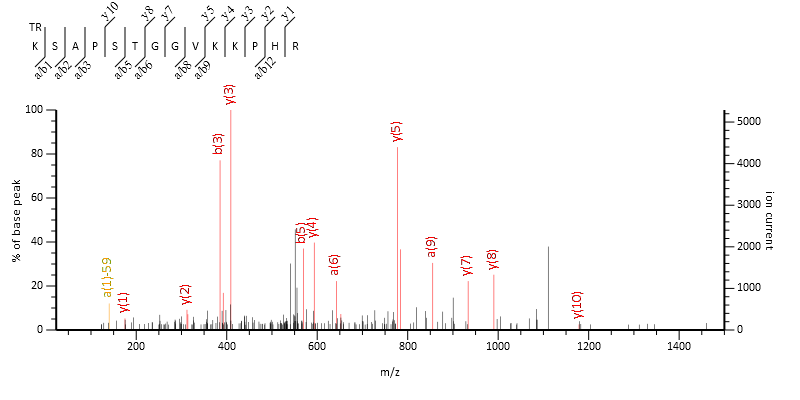

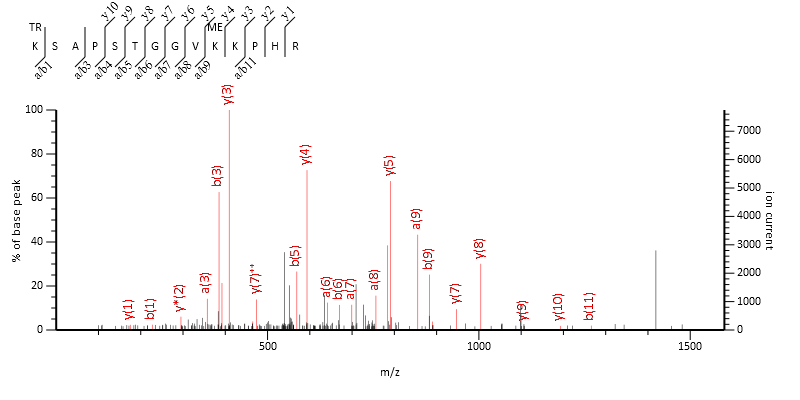

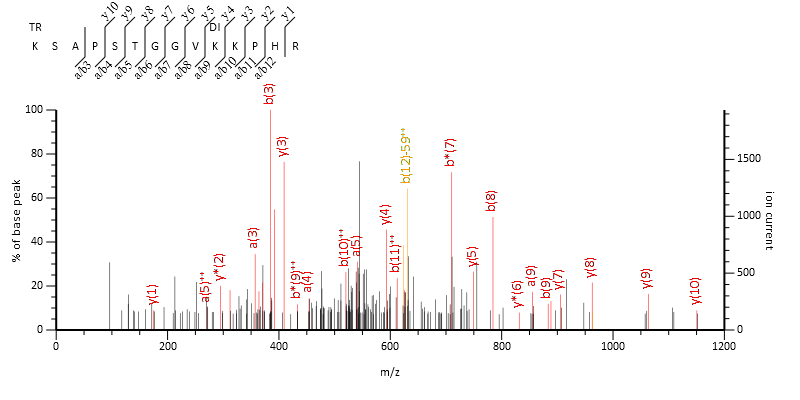

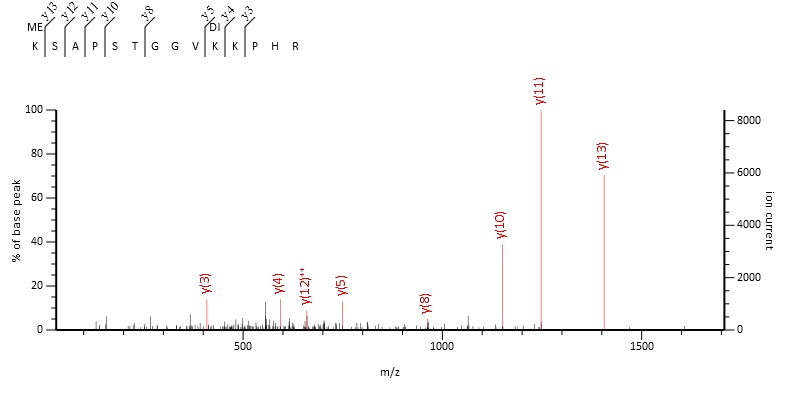

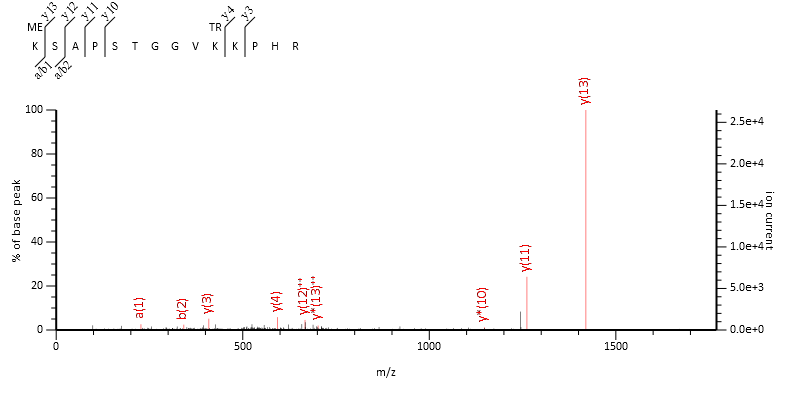

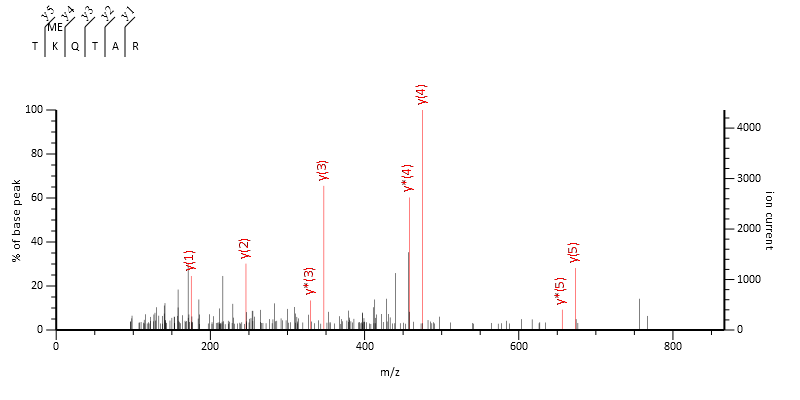

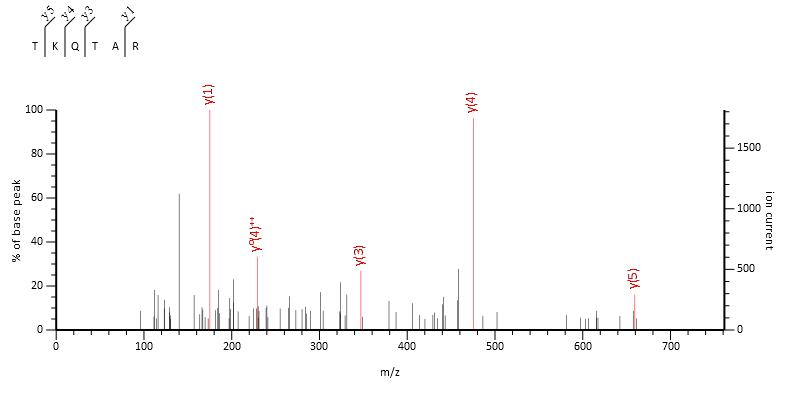

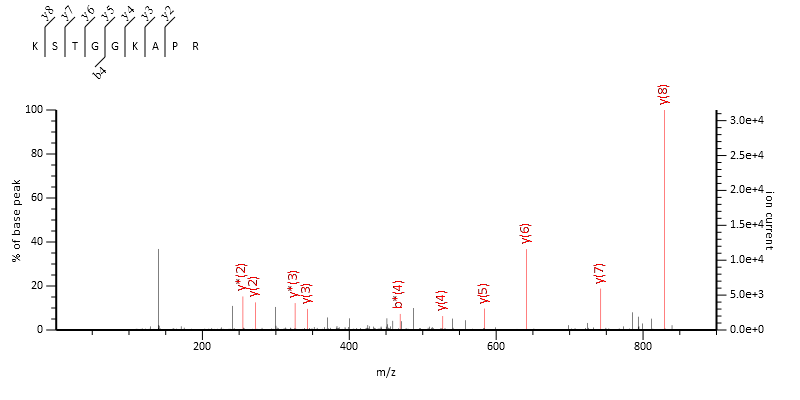

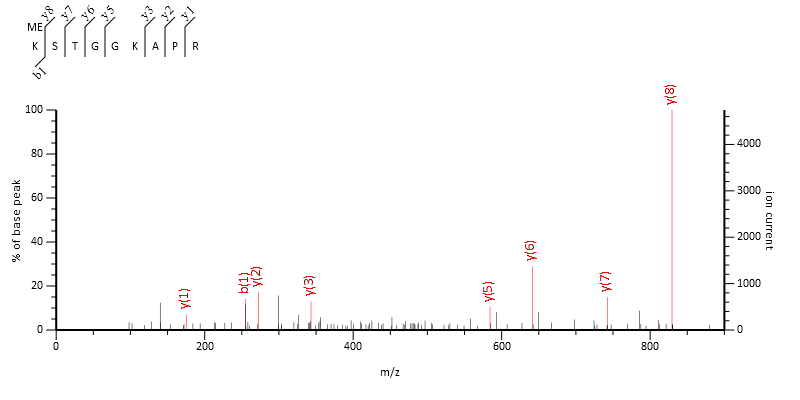

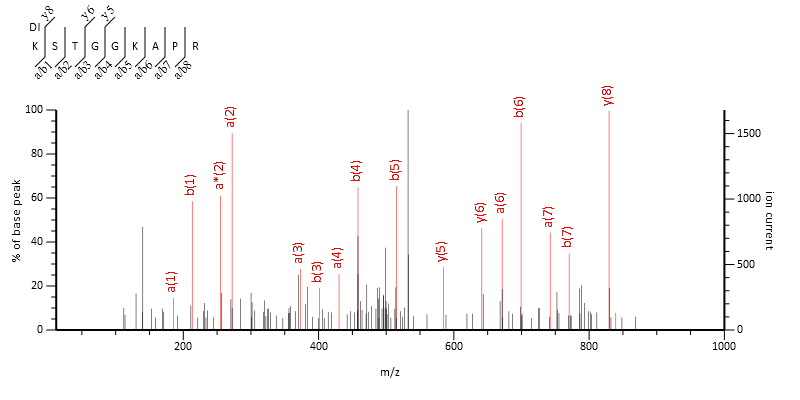

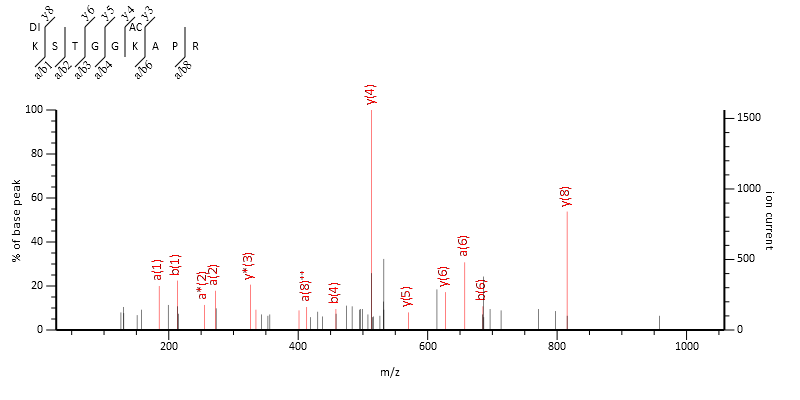

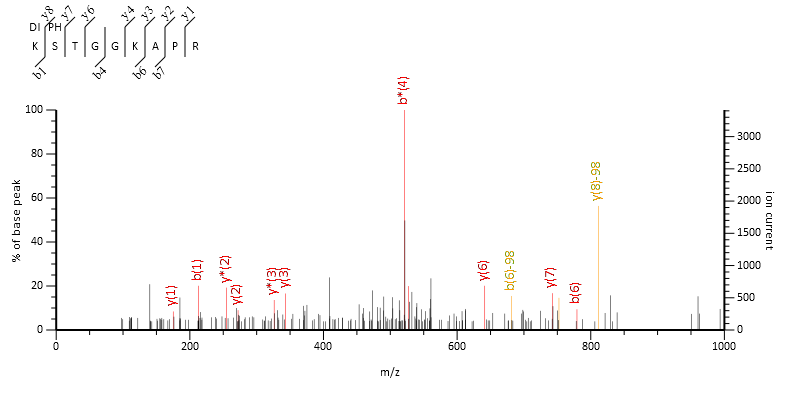

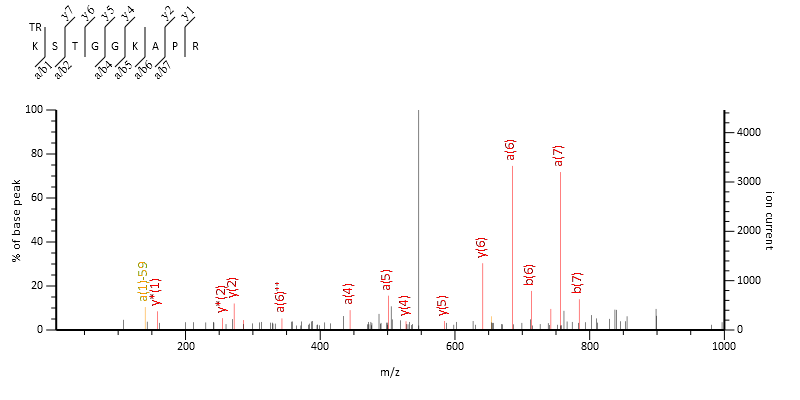

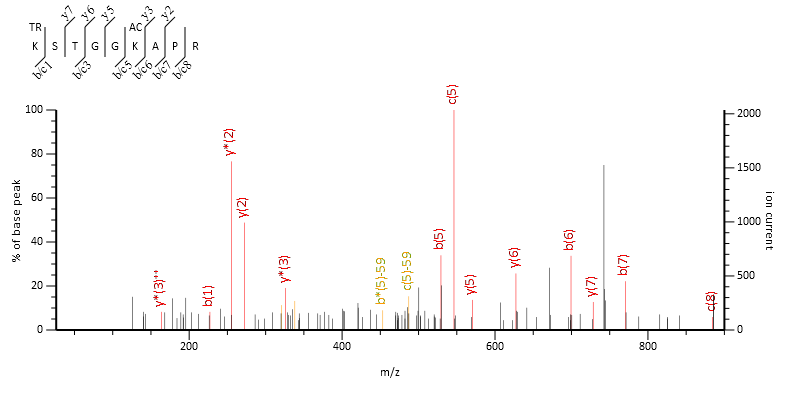

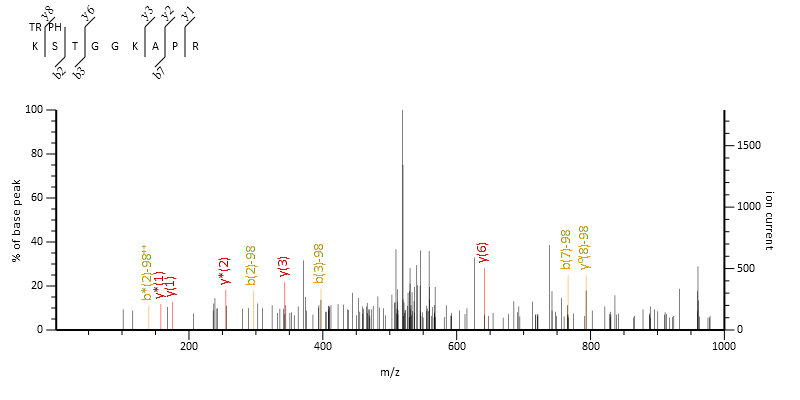

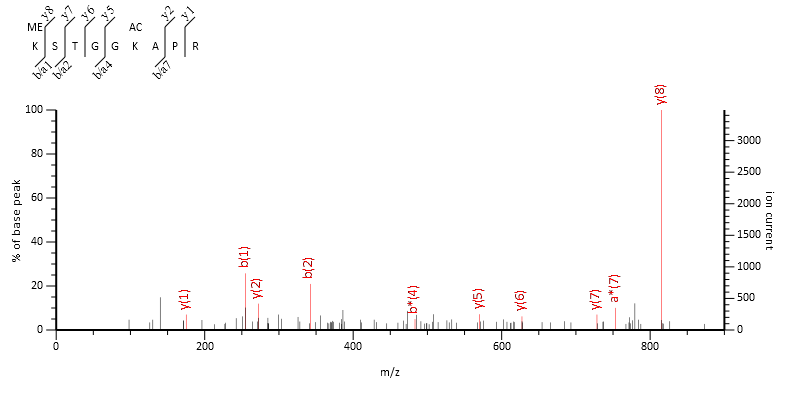

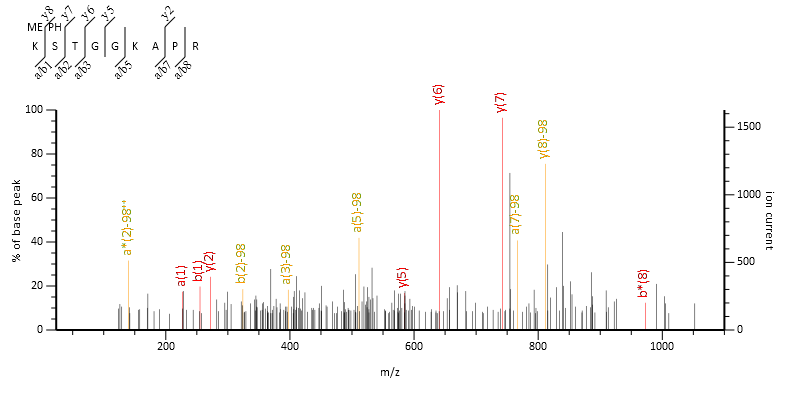

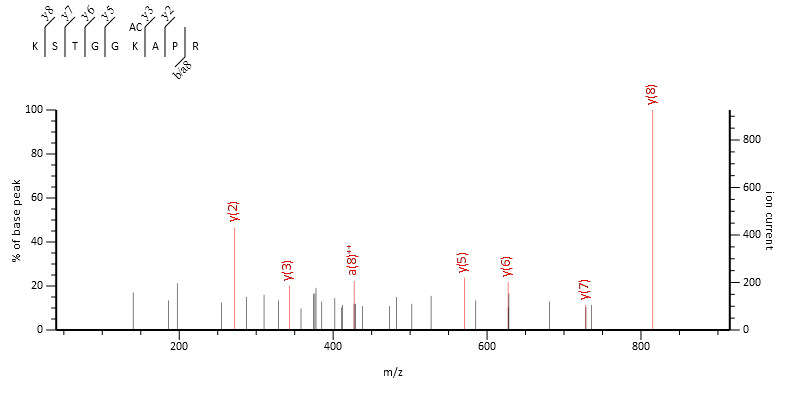

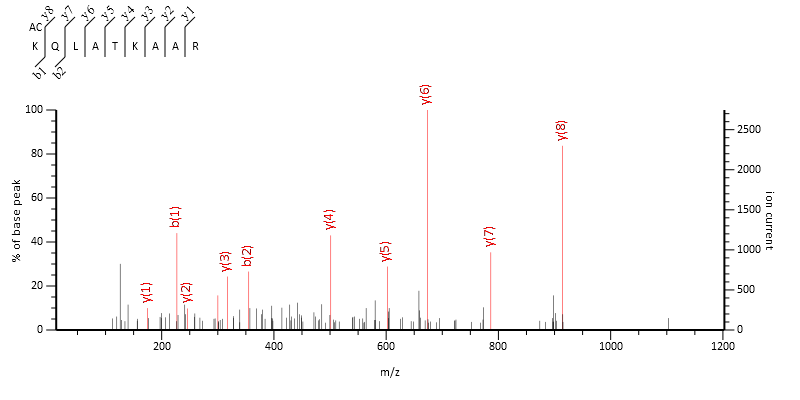

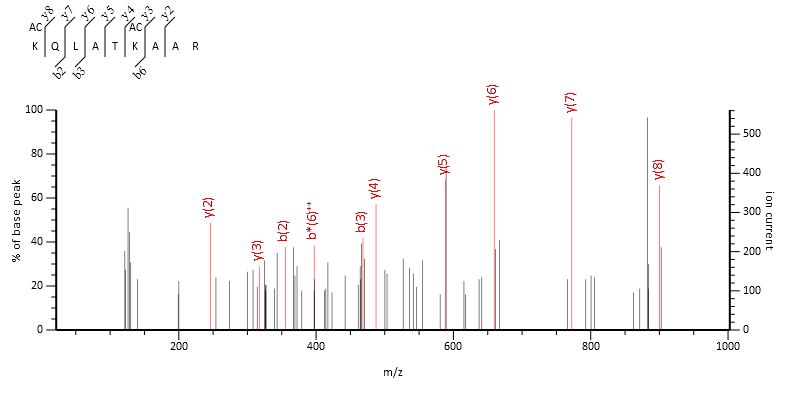

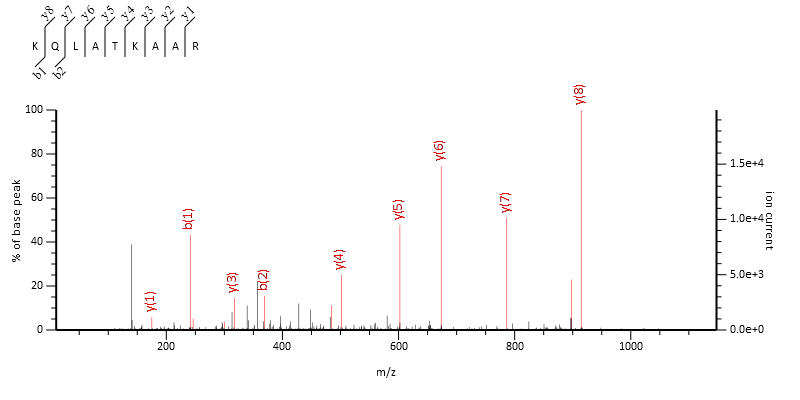

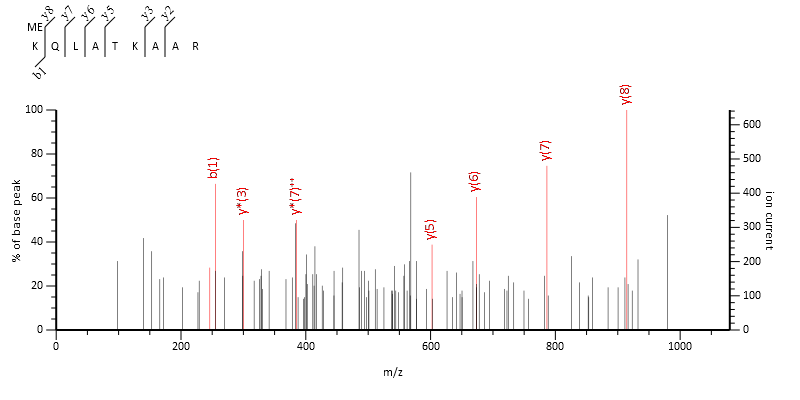

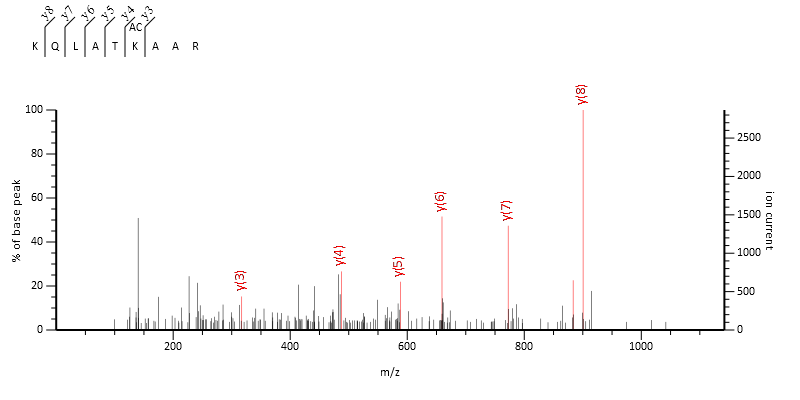

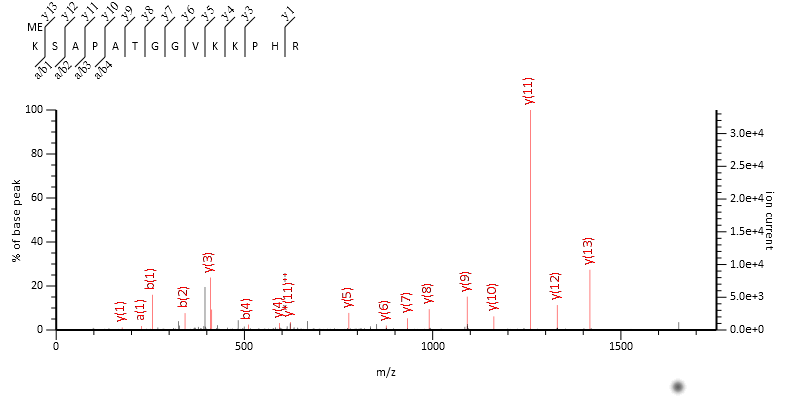

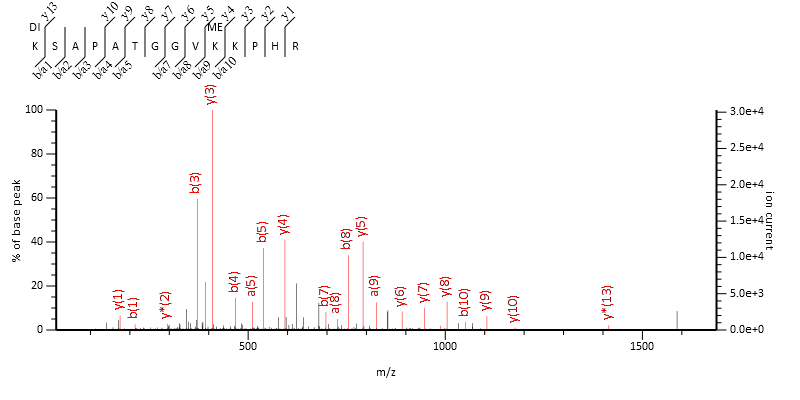

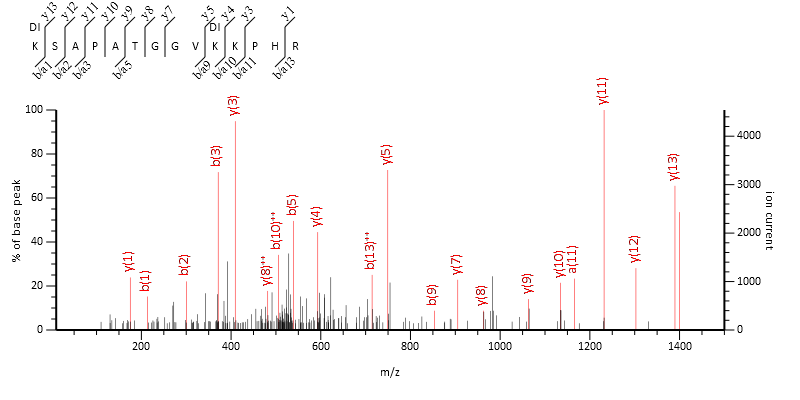

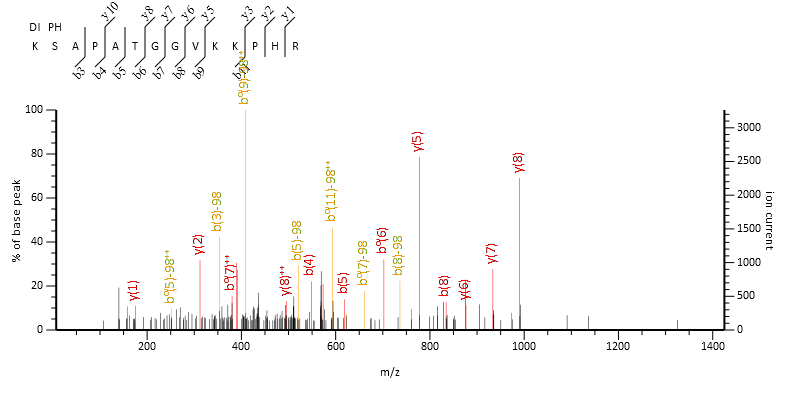

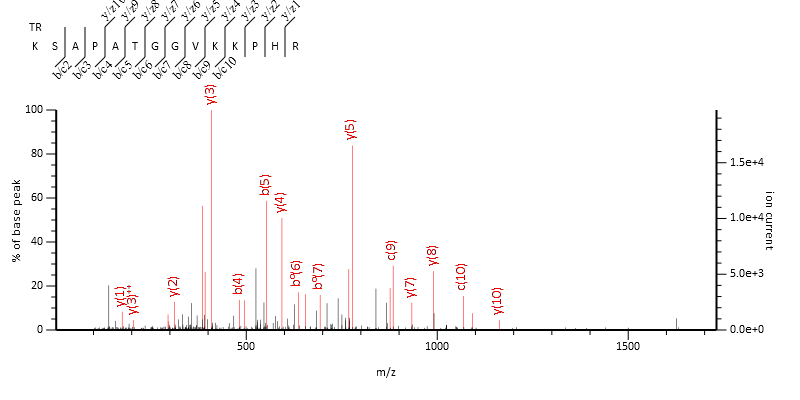

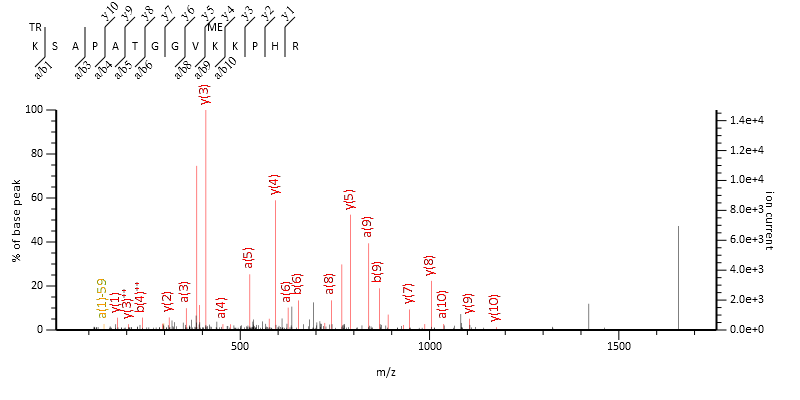

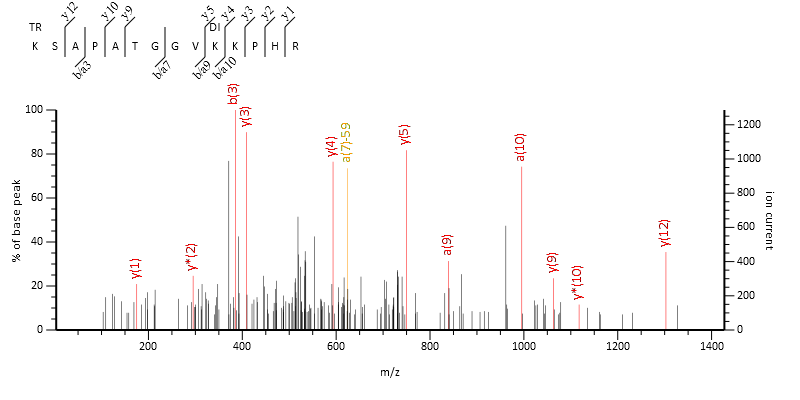

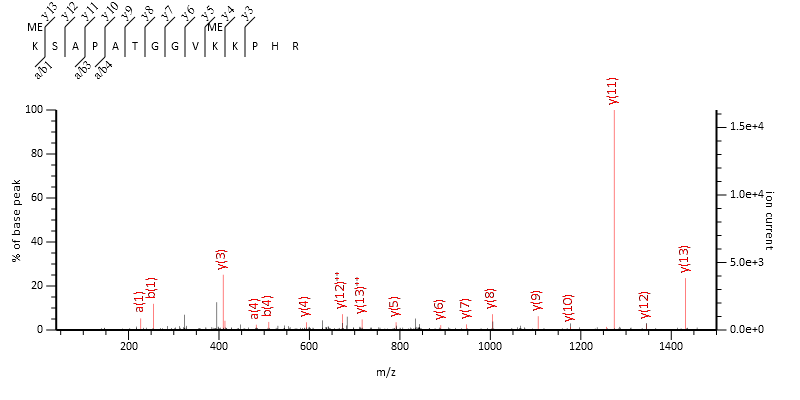

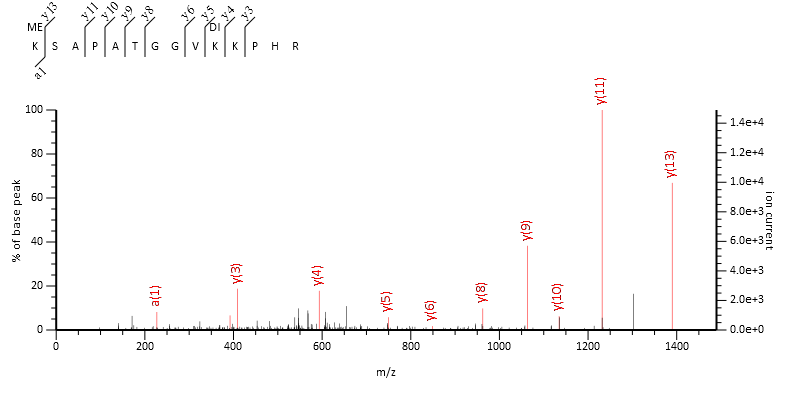

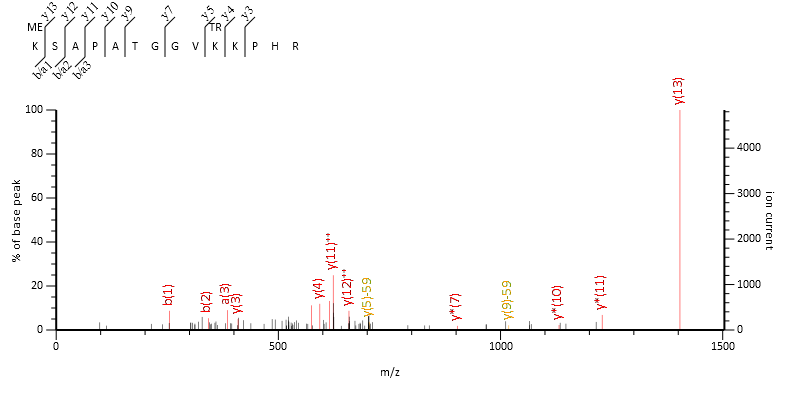

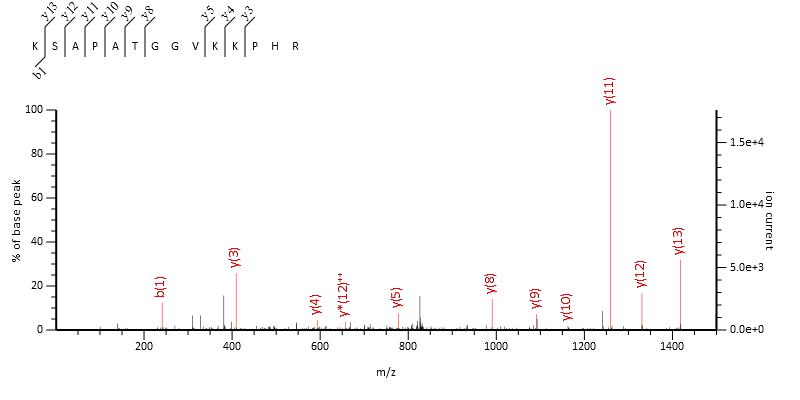

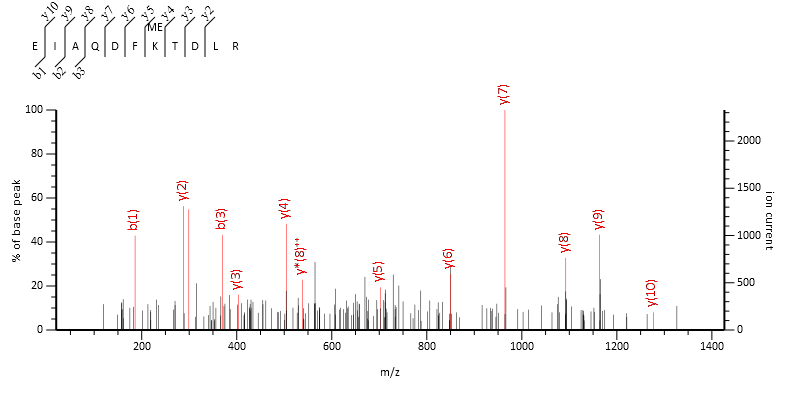

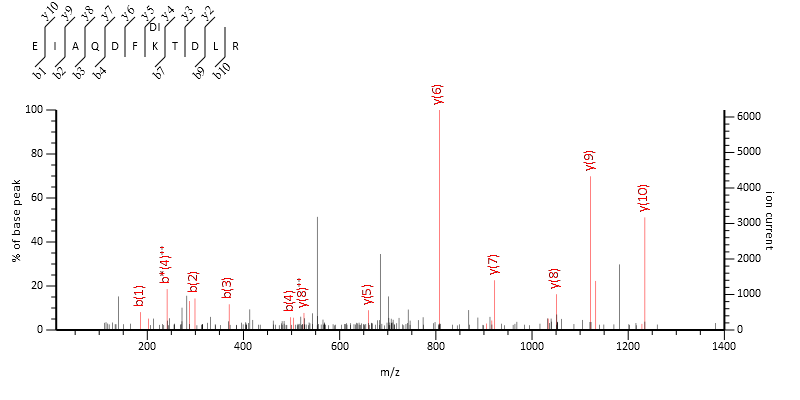

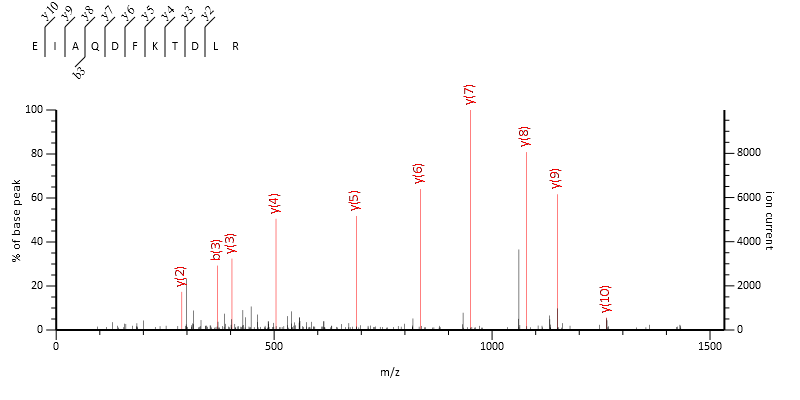

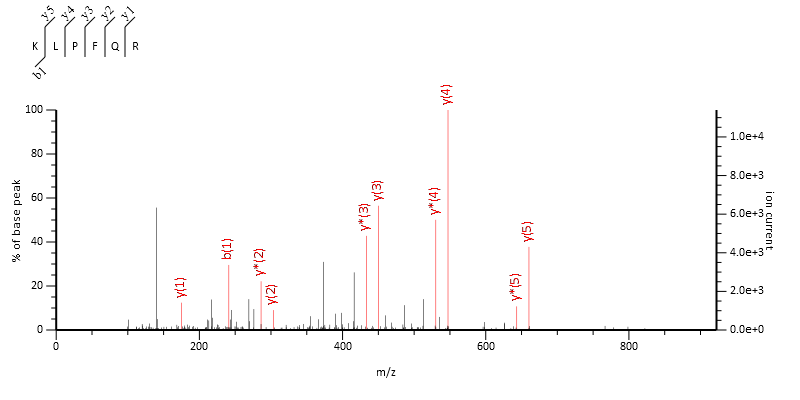

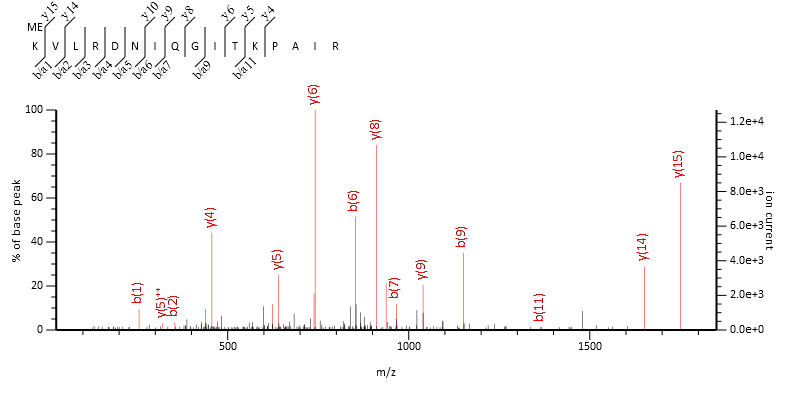

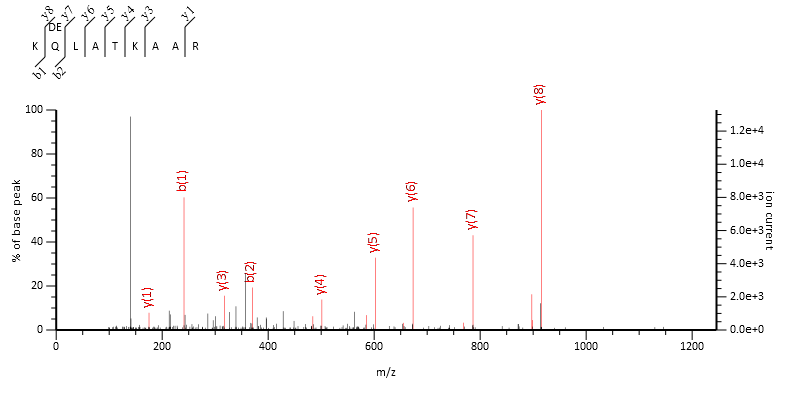

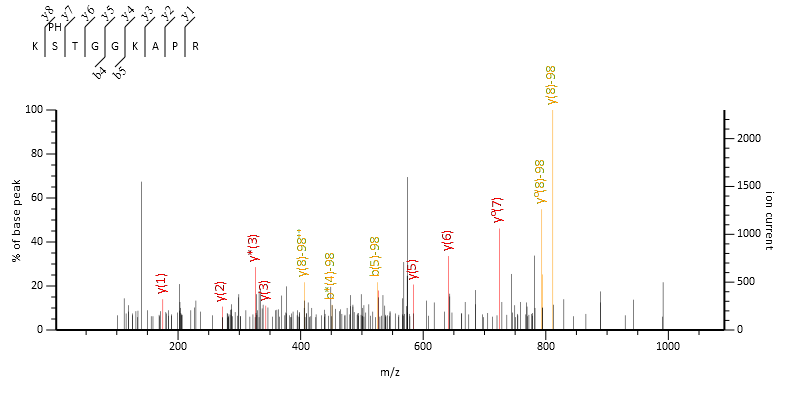

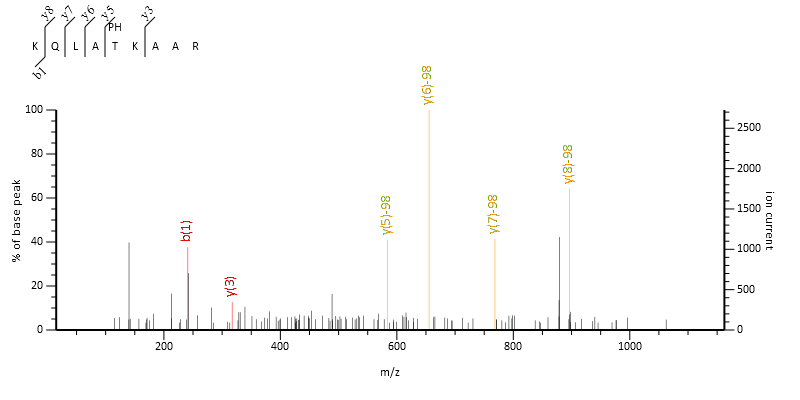

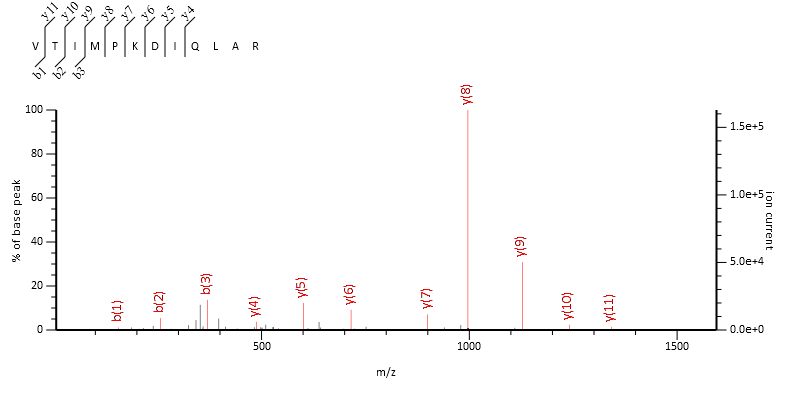

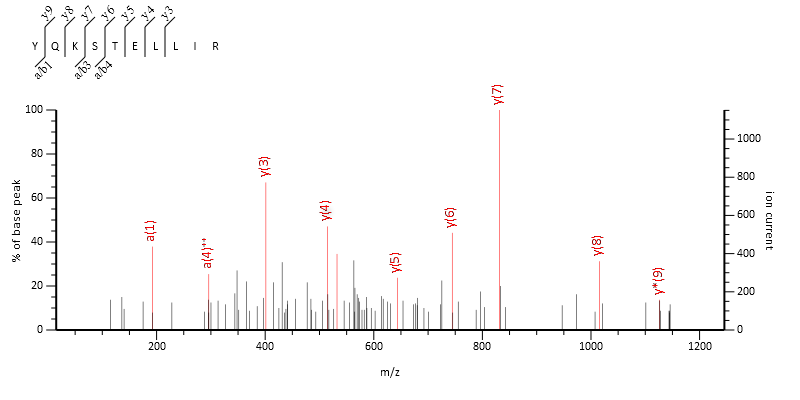

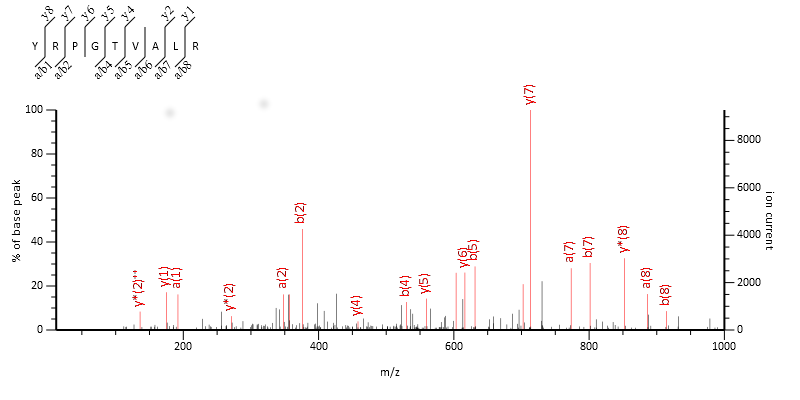

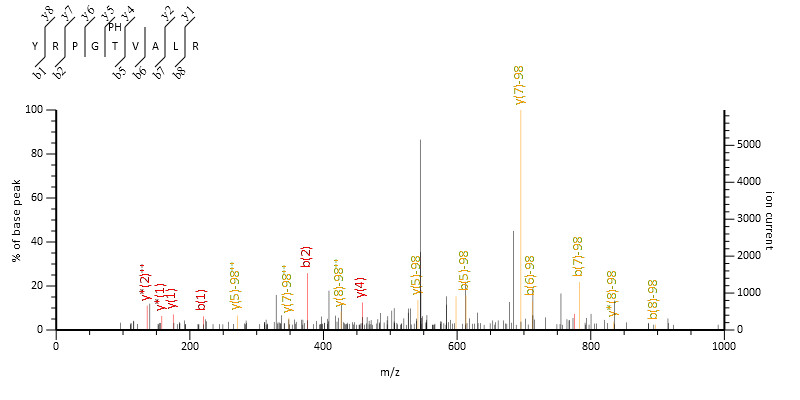
**

**Histone H4 peptides**

**
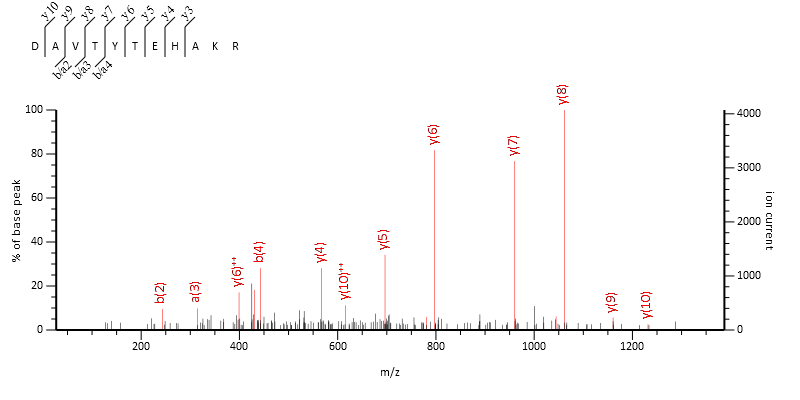

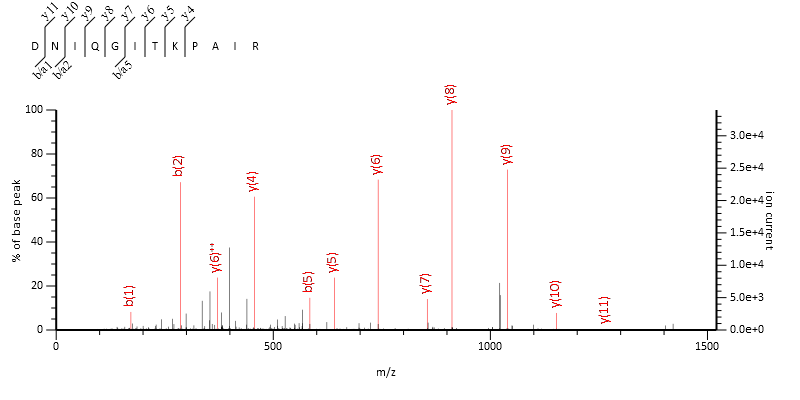

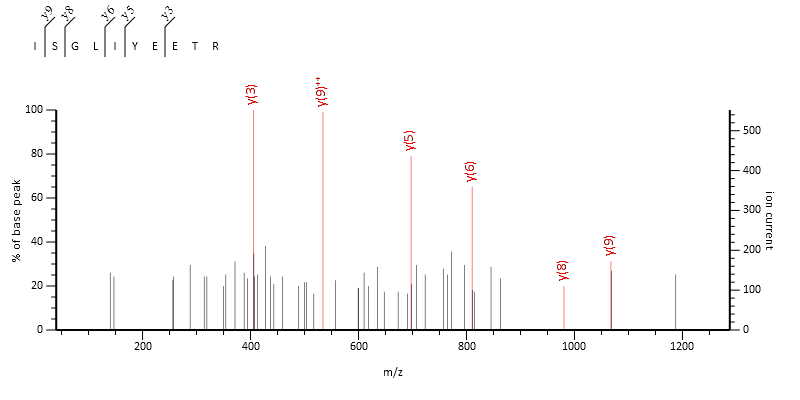

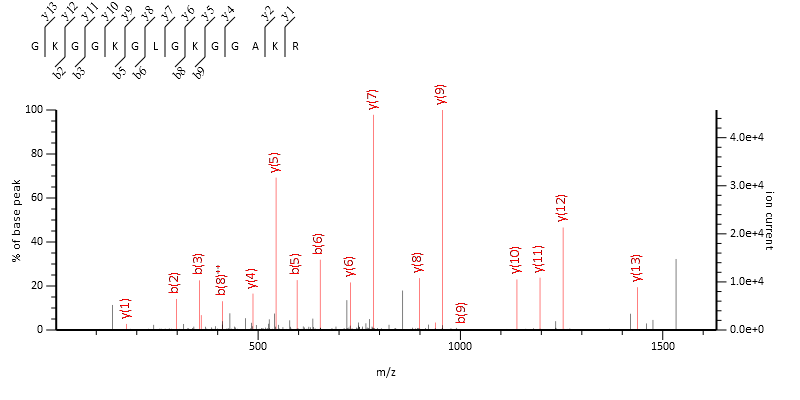

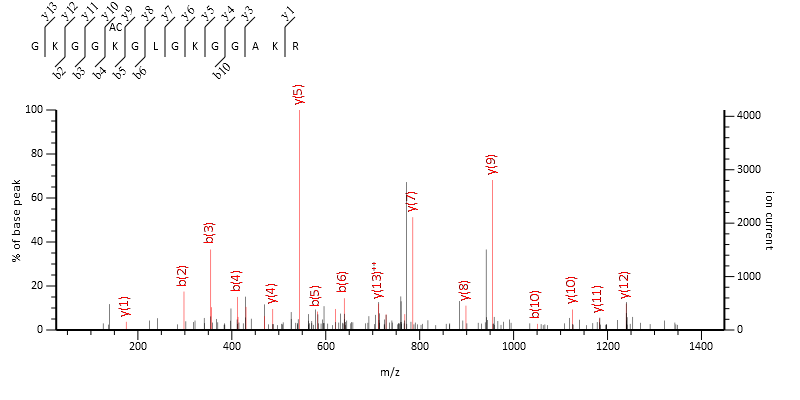

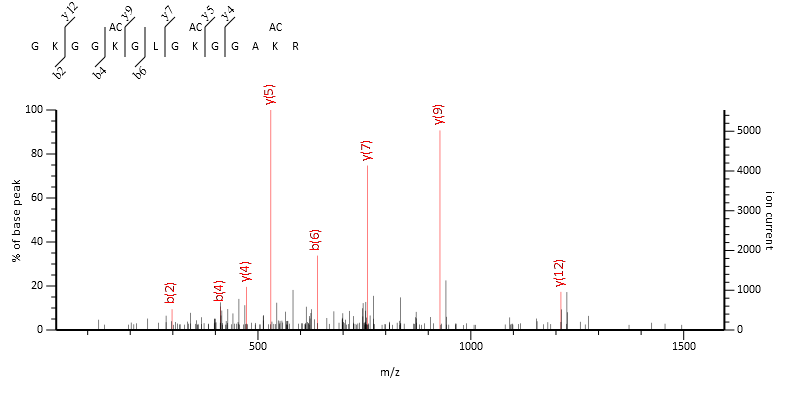

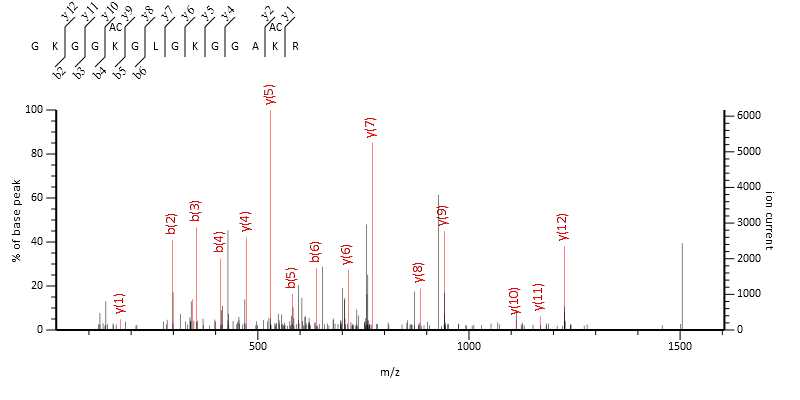

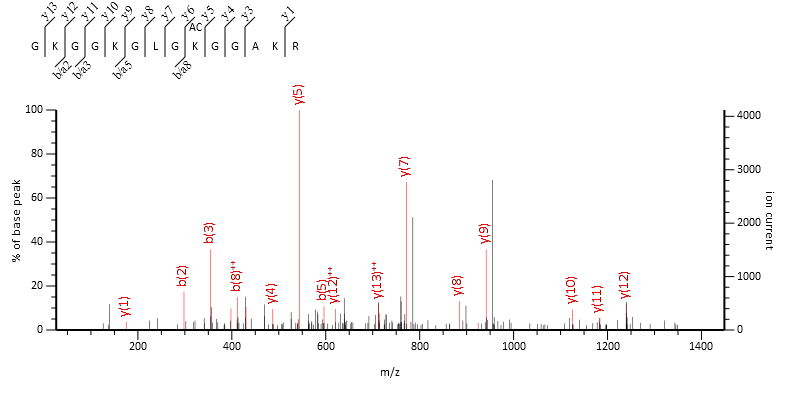

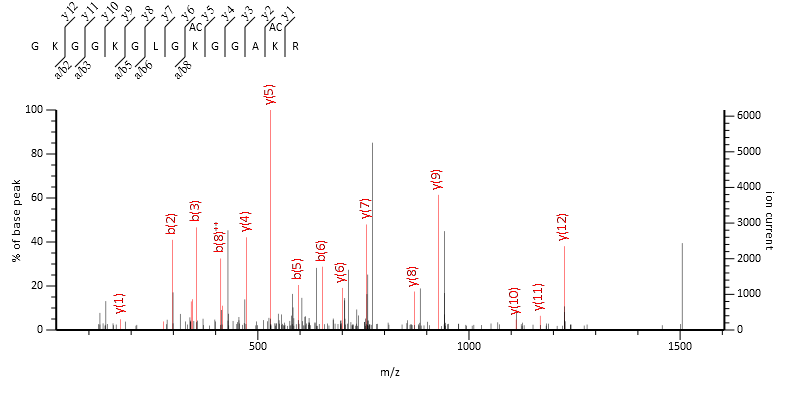

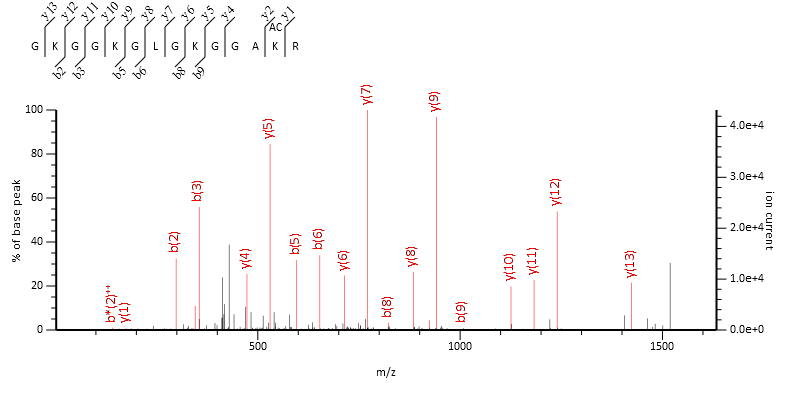

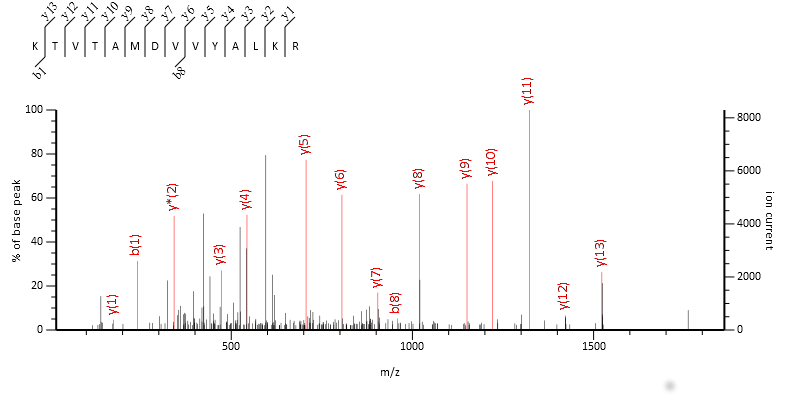

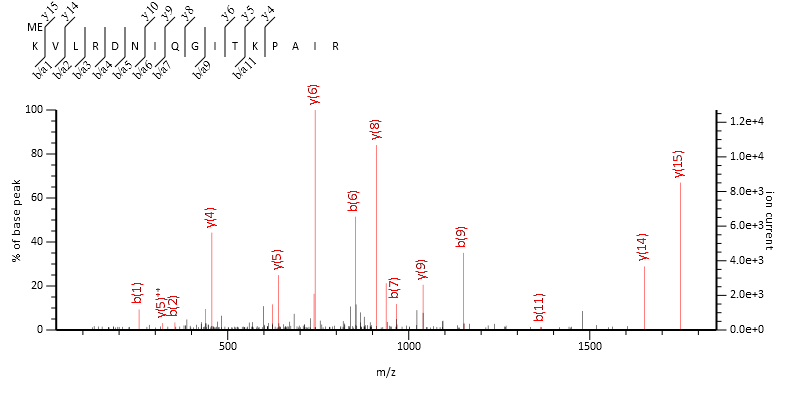

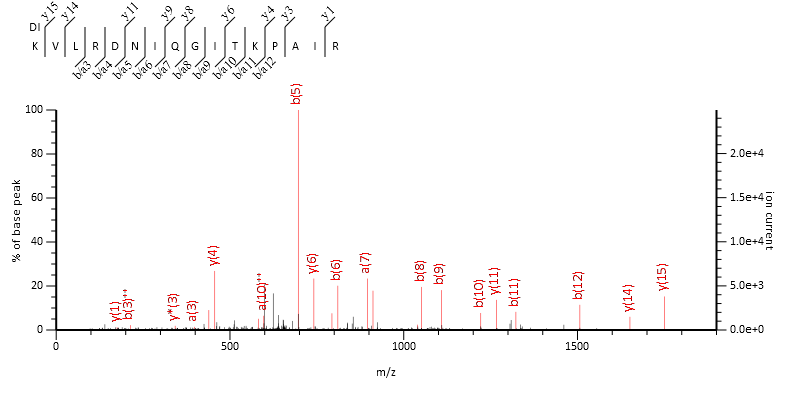
**


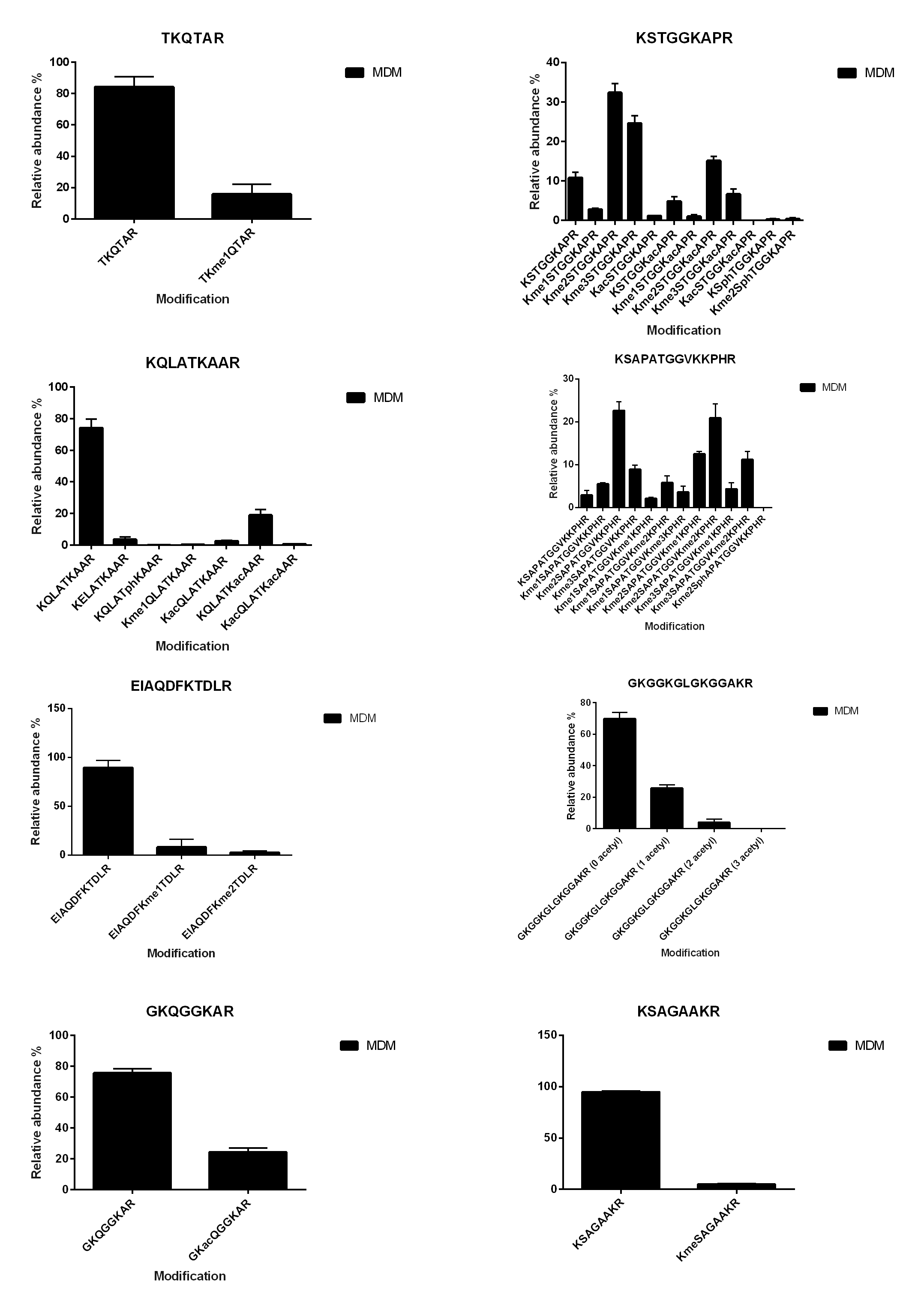


Supplementary Figure 5. Relative quantification of histone post translation modifications from monocyte derived macrophages. Bar charts showing the relative abundance of histone PTMs on peptides from the core histone proteins H1, H2A, H3 and H4 from monocyte derived macrophages. The same monocyte derived macrophage samples were used in the comparison with an n=3. All data is shown as mean with SEM. All data plotted and statistics calculated using GraphPad Prism.
